# Genetic characterization of the *AHAS* mutant line K4 with resistance to AHAS-inhibitor herbicides in rapeseed (*Brassica napus* L.)

**DOI:** 10.1007/s44154-024-00184-8

**Published:** 2025-02-25

**Authors:** Yani Zhang, Qianxin Huang, Shengnan Wang, Lianliang Gao, Gaoping Qu, Yuan Guo, Zhaoxin Hu, Shengwu Hu

**Affiliations:** 1 College of Agronomy, Northwest A&F University, Yangling , Shaanxi, 712100 China; 2 Department of Electrical and Computer Engineering, University of California San Diego, La Jolla, CA 92093 USA

**Keywords:** Rapeseed (*Brassica napus* L.), Acetohydroxyacid synthase (AHAS), Single-point mutation, Herbicides cross-resistance

## Abstract

It remains a great challenge to control weeds in rapeseed fields in China. Breeding herbicide-resistant rapeseed varieties and using corresponding herbicide formulations has become the most economical and effective way to control weeds in rapeseed field. Characterization of more herbicide-resistant genetic resources will provide opportunities for breeders to develop rapeseed herbicide-resistant varieties with good agronomic performance. Previously, we obtained the tribenuron methyl (TBM)-resistant mutant K4 from ZS9 (*Brassica napus* L.) through ethyl methyl sulfonate mutagenesis and TBM foliar-spray screening. In this study, the inheritance and molecular characterization of the mutant K4 are carried out. Genetic investigation indicated that the herbicide-resistance of the K4 was controlled by one dominant allele at a single nuclear gene locus. Molecular characterization showed that a single point substitution at position 535 from C to T in *BnAHAS3* (*BnAHAS3*^*535T*^), which resulted in a mutation at point 179 in BnAHAS3. The K4 showed a certain degree of resistance to TBM, bensulfuron methyl, and monosulfon sodium, which were 50, 30, and 5 times that of ZS9, respectively. AHAS enzyme assay, structural analysis of AHAS proteins, affinity detection between TBM and BnAHAS3 by surface plasmon resonance analysis, and the transgenic experiment in Arabidopsis using *BnAHAS3*^*535T*^ confirmed that *BnAHAS3*^*535T*^ endow the K4 with herbicides resistance. In addition, an allele-specific marker was developed to quickly distinguish the heterozygous and homozygous mutated alleles *BnAHAS3*^*535T*^. In conclusion, our research identified and characterized one novel mutative *AHAS* allele in *B. napus* and enriched genetic resource for developing herbicide-resistant rapeseed cultivars.

**Supplementary Information:**

The online version contains supplementary material available at 10.1007/s44154-024-00184-8.

## Introduction

Rapeseed (*Brassica napus* L., AACC, 2n = 38) is one of the most important oil crops worldwide, particularly in Asia, Europe, North America, and Australia. In China, rapeseed is the top one edible oil crop, covering a plant area of approximately 75 million ha over the last decade (https://faostat.fao.org/). Nowadays, weed management in rapeseed fields has become a critical issue due to the rapid extension of rapeseed mechanized production. Modern agriculture heavily relies on application of herbicides to control weeds. The development of rapeseed varieties with herbicide-resistance and employment of the corresponding herbicide formulations has become the most economical and effective way to control weeds in rapeseed fields (Huang et al. 2020; Guo et al. 2022).

Acetohydroxyacid synthase (AHAS, EC 4.1.3.18), also known as acetolactate synthase (ALS, EC 2.2.1.6), is the key enzyme in the biosynthetic pathway of branched-chain amino acids valine, leucine, and isoleucine (Duggleby and Pang 2000; Mccourt and Duggleby 2006; Duggleby et al. 2008). AHAS is the common target of six categories of herbicides, sulfonylureas (SUs), imidazolinones (IMIs), triazolopyrimidines (TPs), pyrimidinylthiobenzoates (PYBs), sulfonanilides, and sulfonylamino-carbonyltriazolinones (SCTs) (Ray 1984; Shaner et al. 1984; Kleschick et al. 1990; Subramanian et al. 1990; Santel et al. 1999; Hu et al. 2018; Lonhienne et al. 2022). These AHAS-inhibitor herbicides kill susceptible plants by hindering the synthesis of branched-chain amino acids (Duggleby and Pang 2000; Mccourt and Duggleby 2006). They are widely used in wheat or rice fields because of their low dosage, environmentally friendly, low mammalian toxicity, wide crop selectivity, and high efficacy (Heap 2014). However, the availability of AHAS-inhibitor herbicides-resistant rapeseed cultivars is a prerequisite for the utilization of this kind of herbicides in rapeseed fields.

The AHAS enzyme is particular affected by single point mutation or substitution within the highly conservative coding domains (Duggleby et al. 2008). So far, eight single point mutations within AHAS highly conservative domains could reduce plant sensitivity to AHAS-inhibitor herbicides, which included Ala122, Pro197, Ala205, Asp376, Arg377, Trp574, Ser653, and Gly654 (according to the protein sequence of *Arabidopsis thaliana* (APSAT), http://www.weedscience.org/Mutations/MutationDisplayAll.aspx). In *B. napus*, three functional genes *BnAHASs*1-3 have been identified (Rutledge et al. 1991), and several AHAS-inhibitor herbicides-resistant mutants have been obtained through spontaneous or chemical mutagenesis. These mutants included PM1 and M9 that harbor Asp instead of Ser at position 653 of BnAHAS1 APSAT (BnAHAS1^S653A^), PM2 and M342(BnAHAS3^T574L^), PN19 (BnAHAS1^T574L^), M45(BnAHAS3^P197L^), M196 (BnAHAS1^P197L^), and K5 (BnAHAS1^P197S^) (Swanson et al. 1989; Tonnemaker et al. 1992; Hattori et al. 1995; Hu et al. 2015; Li et al. 2015; Huang et al. 2020; Guo et al. 2020, 2022). Recently, two double-loci mutants have been reported, the mutant 5N (BnAHAS1^T574L^ and BnAHAS3^T574L^) was developed through pyramiding two mutant genes of PN19 and M342 by molecular marker-assisted selection (Guo et al. 2020), and DS3 (BnAHAS3 ^T574L^ and BnAHAS1^P197L^) by two rounds of ethyl methane sulfonate (EMS) mutagenesis (Guo et al. 2022). These AHAS mutants have been used to develop herbicide-resistant rapeseed varieties (Tan et al. 2005; Guo et al. 2020) and are recommended to be used as male parents in chemical induced male sterility hybrid seed production (Li et al. 2015; Lv et al. 2018). In addition, by transforming the *csr-1* gene from Arabidopsis, the SU-resistant rapeseed with the P197H mutation was created (Blackshaw et al. 1994). Using the CRISPR/Cas-base editing technology, the amino acid substitutions of P197F/S (APSAT) in BnAHAS1, BnAHAS3, or both BnAHAS1 and BnAHAS3 were generated in rapeseed with an SU-resistant phenotype (Wu et al. 2020; Cheng et al. 2021). The availability of more herbicide-resistant genetic resources will provide opportunities for breeders to develop rapeseed varieties with herbicide-resistance and good agronomic performance in different rapeseed growing regions.

In previous studies, we obtained three tribenuron-methyl (TBM)-resistant mutants (K1, K4, and K5) derived from Zhongshuang No.9 (ZS9) via EMS mutagenesis and TBM foliar-spray screening (Qu et al. 2014; Sun et al. 2015), and characterized the mutant K5 (Lv et al. 2018; Huang et al. 2020). In this study, the TBM-resistant rapeseed mutant K4 is characterized. The objectives of the present study are: (1) to reveal the resistance or cross-resistance of K4 to different AHAS-inhibitor herbicides; (2) to figure out the mode of inheritance of its herbicide resistance; and (3) to dissect the molecular mechanism of its herbicide resistance. The findings will lay foundation for developing rapeseed varieties with herbicide-resistance.

## Results

### Cross-resistance of the mutant line K4 to different herbicides

**Fig. 1:**
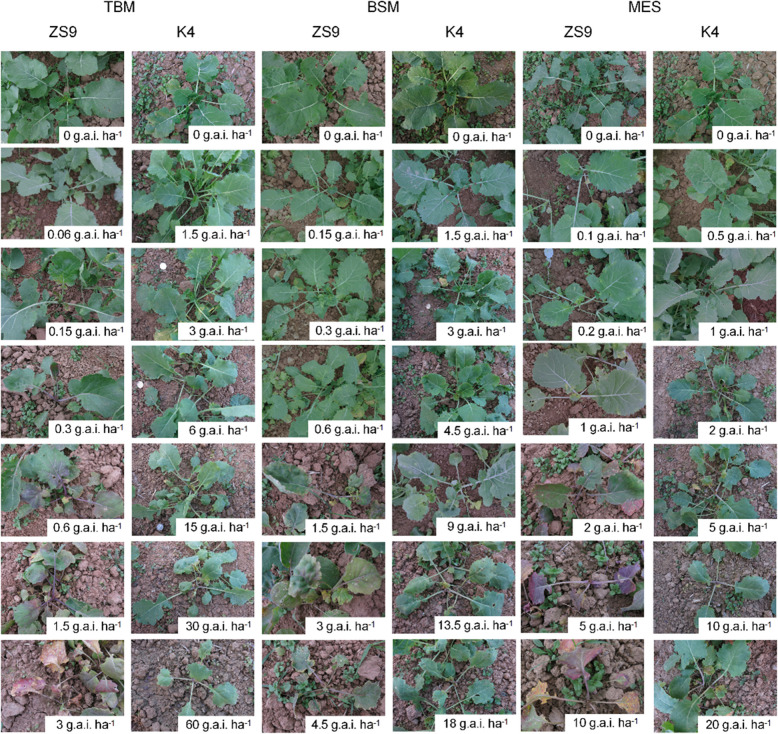
Phenotype of K4 and ZS9 plants three weeks after being sprayed with three different herbicides. TBM, tribenuron-methyl; BSM, bensulfuron-methyl; MES, monosulfuron sodium; ZS9, Zhongshuang No.9; K4, the mutant line; g. a.i. ha^−1^, gram active ingredients hectare^−1^

In order to investigate the herbicide response of the mutant line K4, at the five-leaf stage, seedlings of K4 as well as wild type (WT) ZS9 were foliar sprayed with different rates of four herbicides, TBM, bensulfuron-methyl (BSM), monosulfuron-sodium (MES), and florasulam (FU) (Table 1). The herbicides-resistance test revealed that ZS9 was susceptive to almost all application rates of the four herbicides (Fig. 1). However, the K4 showed a certain degree of resistance to three herbicides TBM, BSM, and MES (Fig. 1), but susceptive to FU.

**Table 1:** Herbicides and their rates in a volume of 300 L ha^−1^ used for cross-resistance test for the lines ZS9 and K4 in *Brassica napus* L

Herbicides	Rates for ZS9 (g a.i. ha^−1^)	Rates for K4 (g a.i. ha^−1^)
Tribenuron-methyl	0	0
	0.06	1.5
	0.15	3.0
	0.3	6.0
	0.6	15.0
	1.5	30.0
	3.0	60.0
Bensulfuron-methyl	0	0
	0.15	1.5
	0.3	3.0
	0.6	4.5
	1.5	9.0
	3.0	13.5
	4.5	18.0
Florasulam	0	0
	0.6	3.0
	1.5	6.0
	3.0	9.0
		15.0
Monosulfon- sodium	0	0
	0.1	0.5
	0.2	1.0
	0.5	2.0
	1.0	5.0
	2.0	10.0
	5.0	20.0
	10.0	

ZS9 Zhongshuang No.9, K4 the mutant line; g a.i. ha^−1^, gram active ingredients hectare^−1^

The K4 exhibited a relatively high resistance to TBM (Fig. 1). Three weeks after foliar-spraying TBM, no apparent phytotoxicity was observed for ZS9 plants treated with 0.06 g active ingredients per hectare (g. a.i. ha^−1^) TBM, however, a variety of symptoms were observed for ZS9 plants treated with the rest rates of TBM, and all the plants dead under treatment of 0.6 g a.i. ha^−1^ rate of TBM (Fig. 1). For the K4, the plants treated with the TBM rates ≤ 3.0 g a.i. ha^−1^ did not show significant symptoms; the plants treated with the TBM rate of 6.0 g a.i. ha^−1^ showed symptoms on newly growing leaves and stems; the plants treated with the TBM rate of 15 g a.i. ha^−1^ were injured badly; the growing points of plants treated with the TBM rate of 60 g a.i. ha^−1^ were inhibited, and all the plants at this rate dead over winter (Fig. 1), indicating that the TBM-lethal rate for K4 was 50–100 times that of ZS9 (Table 2). Meanwhile, five indices (phytotoxicity index, leaf angle, leaf numbers, fresh weight, and dry weight) were investigated for all the treatments, and the results showed that, for ZS9, the herbicide-free and 0.06 g a.i. ha^−1^ TBM treatments showed no significant difference in the five indices, which indicated that 0.06 g a.i. ha^−1^ was the safety rate of TBM for ZS9; however, the safety rate of TBM for K4 was 3.0 g a.i. ha^−1^ according to five indices, indicating that the TBM safety rate for K4 was approximately 50 times that of ZS9 (Table 2, Tables S3, S4). Additionally, the* I*_*50*_ of fresh weight and dry weight of K4 were also approximately 50 times that of ZS9 (Table 2). Therefore, the TBM safety rate for K4 was 3.0 g a.i. ha^−1^, the lethal rate for K4 ranged from 30 g a.i. ha^−1^ to 60 g a.i. ha^−1^, the TBM-resistance of K4 was approximately 50 times that of ZS9 (Table 2).

**Table 2:** Cross-resistance of the mutant line K4 to three different herbicides

Herbicides	Lines	*I*_*50*_ (g a.i. ha^−1^)		Lethal rate (g a.i. ha^−1^)	Safety rate (g a.i. ha^−1^)
		Fresh weight	Dry weight		
Tribenuron-methyl	ZS9	0.36 ± 0.06	0.30 ± 0.06	0.60	0.06
	K4	13.32 ± 7.11^a^	10.32 ± 3.00^a^	30.0 ~ 60.0	3.00
Bensufuron-methyl	ZS9	0.27 ± 0.12	0.30 ± 0.15	1.50	0.15
	K4	10.98 ± 1.00^a^	8.37 ± 3.33^a^	> 18.00	4.50
Monosufuron sodium	ZS9	0.39 ± 0.12	0.36 ± 0.12	1.00	0.10
	K4	1.64 ± 0.31^a^	1.47 ± 0.48^a^	> 20.00	1.00

ZS9 Zhongshuang No.9, K4 the mutant line; *I*_*50*_ is the herbicide rate required to reduce fresh/dry weight by 50%

^a^indicated a significant difference between ZS9 and K4 in the tested traits at *p* = 0.01 level; g a.i. ha^−1^, gram active ingredients hectare^−1^

The K4 showed a certain degree resistance to BSM and MES (Table 2, Tables S3, S4, and Fig. 1). The BSM-lethal rate for K4 was > 18 g a.i. ha^−1^, the BSM-lethal rate for ZS9 was 1.5 g a.i. ha^−1^, the safety rate of BSM for K4 (4.5 g a.i. ha^−1^) was 30 times that of ZS9 (0.15 g a.i. ha^−1^), and the *I*_*50*_ of fresh weight and dry weight of K4 were also approximately 30 times that of ZS9 (Table 2). Therefore, the BSM-resistance of K4 was approximately 30 times that of ZS9. The MES-lethal rate for K4 was > 20 g a.i. ha^−1^, the MES-lethal rate for ZS9 was 1.0 g a.i. ha^−1^, the safety rate of MES for K4 (1.0 g a.i. ha^−1^) was 10 times that of ZS9 (0.10 g a.i. ha^−1^), and the *I*_*50*_ of fresh weight and dry weight of K4 were also approximately 5 times that of ZS9 (Table 2). Therefore, the MES-resistance of K4 was approximately 5 times that of ZS9.

In conclusion, the K4 showed a certain degree resistance to the three herbicides TBM, BSM, and MES, which was 50, 30, and 5 times that of ZS9, respectively.

### AHAS enzyme activity assay

**Fig. 2:**
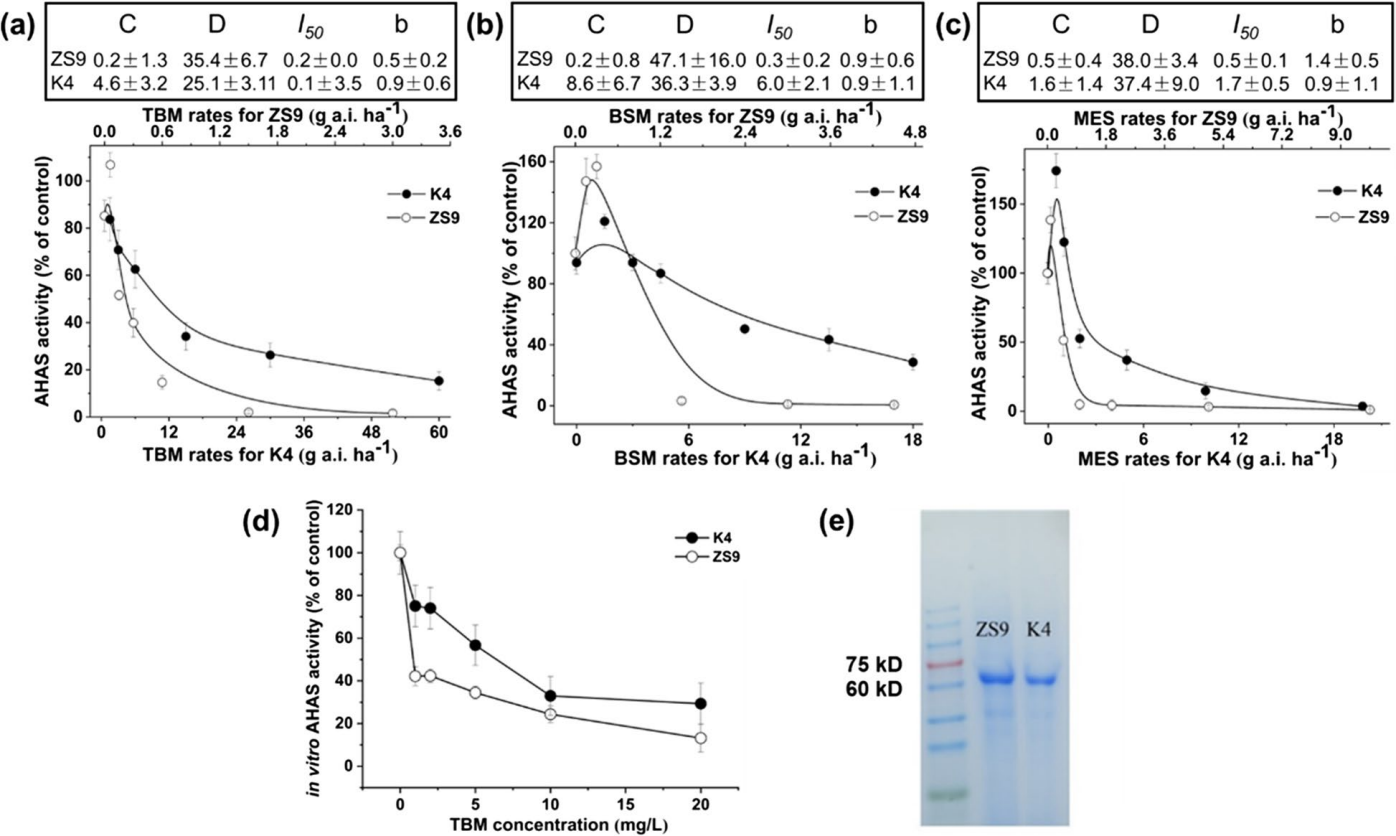
Changes in AHAS relative activities of rapeseed ZS9 and the mutant K4 after being sprayed with three kinds of herbicides. **a**-**c**, Relative AHAS activity of ZS9 and K4 after being sprayed with tribenuron-methyl (TBM) (**a**), bensufuron-methyl (BSM) (**b**), and monosulfuron-ester sodium (MES) (**c**). **d**, In vitro relative AHAS activity of rapeseed ZS9 and the mutant K4 treated with different concentrations of TBM. **d**, Sodium dodecyl sulfate-polyacrylamide gel electrophoresis (SDS-PAGE) analysis of AHAS enzyme extracted from fresh leaves of herbicide-free K4 and ZS9.C and D are the lower and upper asymptotes, respectively, of the AHAS activity; *I*_*50*_ is the herbicide dose required to reduce the AHAS activity by 50% and *b *is the slope of the curve at approximately *I*_*50*_. ZS9, Zhongshuang No.9; K4, the mutant line. In the absence of herbicides, no significant difference was detected in AHAS average specific activity (U mg^-1^ protein h^-1^) between K4 (0.084 ± 0.007, *n *=4) and ZS9 (0.098 ± 0.013, *n *=4)

To figure out whether the herbicides-resistance of the K4 is the result of the gain-of-function mutation of AHAS, the enzyme activity of seedlings of the K4 and ZS9 was detected after treated with different rates of the three herbicides TBM, BSM, and MES. The molecular weight of AHAS isolated from K4 and ZS9 seedlings was approximately 58–66 kD (Fig. 2e), AHAS specific activity of ZS9 seedlings was 0.098 ± 0.011 unit.milligram^−1^.hour^−1^ (U.mg^−1^.h^−1^), and that of K4 seedlings was 0.084 ± 0.007 U.mg^−1^.h^−1^, there did not exist significant difference between them (*t* = 3.30, *p* = 0.19), which indicated that the herbicides-resistance of K4 was not caused by the increase in AHAS activity. Three weeks after spraying with TBM, the relative AHAS activity of ZS9 dropped rapidly with the increase of TBM rate; however, that of K4 reduced slowly with the increase of TBM rate. The relative AHAS activity of K4 could be inhibited by 50% (*I*_*50*_) with 10.1 g a.i. ha^−1^ TBM, which was approximately 50 times that of ZS9 (*I*_*50*_ = 0.2 g a.i. ha^−1^) (Fig. 2a). The AHAS activity of K4 and ZS9 seedlings treated with BSM or MES, changed similarly as that treated with TBM (Fig. 2b and c). The AHAS activity of K4 could be inhibited by 50% (*I*_*50*_) with the BSM and MES rates at 6.0 g a.i. ha^−1^ and 1.7 g a.i. ha^−1^, respectively, which were approximately 20 times and 3 times that of ZS9 (0.3 g a.i. ha^−1^ and 0.5 g a.i. ha^−1^), respectively (Fig. 2b and c). Moreover, the in vitro AHAS activity of both herbicide-free K4 and ZS9 seedlings was also detected, and the in vitro AHAS activity of ZS9 was inhibited by 40% at the TBM concentration of 1 mg. L^−1^, however, for the K4, the TBM concentration of 10 mg. L^−1^ was required to do this work (Fig. 2d). In conclusion, the herbicides-resistance of the K4 is the result of the gain-of-function mutation of AHAS, but not the overexpression of AHAS.

### Genetic investigation of TBM-resistance of the mutant line K4

The mutant line K4, two susceptive lines SH11 and ZS9, and their derived progenies F_1_, BC_1_, and F_2_ seedlings were foliar-sprayed with 4.5 g a.i. ha^−1^ TBM at the 4–6 leaf stage to investigate inheritance of the herbicide-resistance in K4. The results showed that all the F_1_ individuals represented a certain degree of TBM-resistance, indicating that the resistance was controlled by dominated gene(s) (Table 3). The F_2_ population derived from the cross between K4 and ZS9 segregated in a ratio of 1295 resistant plants: 397 susceptible plants. The BC_1_ population segregated in a ratio of 46 resistant plants: 38 susceptible plants. A goodness-of-fit test indicated that the segregation ratio of resistant to susceptible plants fits the expected Mendelian ratios of 3:1 and 1:1 (χ^2^_c_ = 0.38, *p* = 0.54 and χ^2^_c_ = 1.00, *p* = 0.30; Table 3), respectively. The F_2_ and BC_1_ populations derived from the cross between K4 and SH11 also showed similar results (χ^2^_c_ = 0.32, *p* = 0.57 and χ^2^_c_ = 0.12, *p* = 0.73; Table 3). Generally, the results indicated that the herbicide- resistance in the K4 was controlled by one pair of nuclear genes, which had dominant resistance.

**Table 3:** Inheritance of the tribenuron-methyl resistance in the mutant line K4

Cross and populations	Resistant plants	Sensitive plants	Expected ratio	Ratio	χ^2^_c_	*P*
**K4 × ZS9**						
F_1_	99	0	-	-	-	-
BC_1_	46	38	1:1	1.2:1	0.38	0.54
F_2_	1295	397	3:1	3.2:1	1.00	0.30
**K4 × SH11**						
F_1_	28	0	-	-	-	-
BC_1_	260	242	1:1	1.07:1	0.32	0.57
F_2_	697	241	3:1	2.9:1	0.12	0.73

χ^2^_0.05,1_ = 3.84, χ^2^_0.01,1_ = 6.63

ZS9 Zhongshuang No.9, K4 the mutant line; -, not applied

### Comparative sequencing of *BnAHASs1-3* of the mutant K4 and wild type

**Fig. 3:**
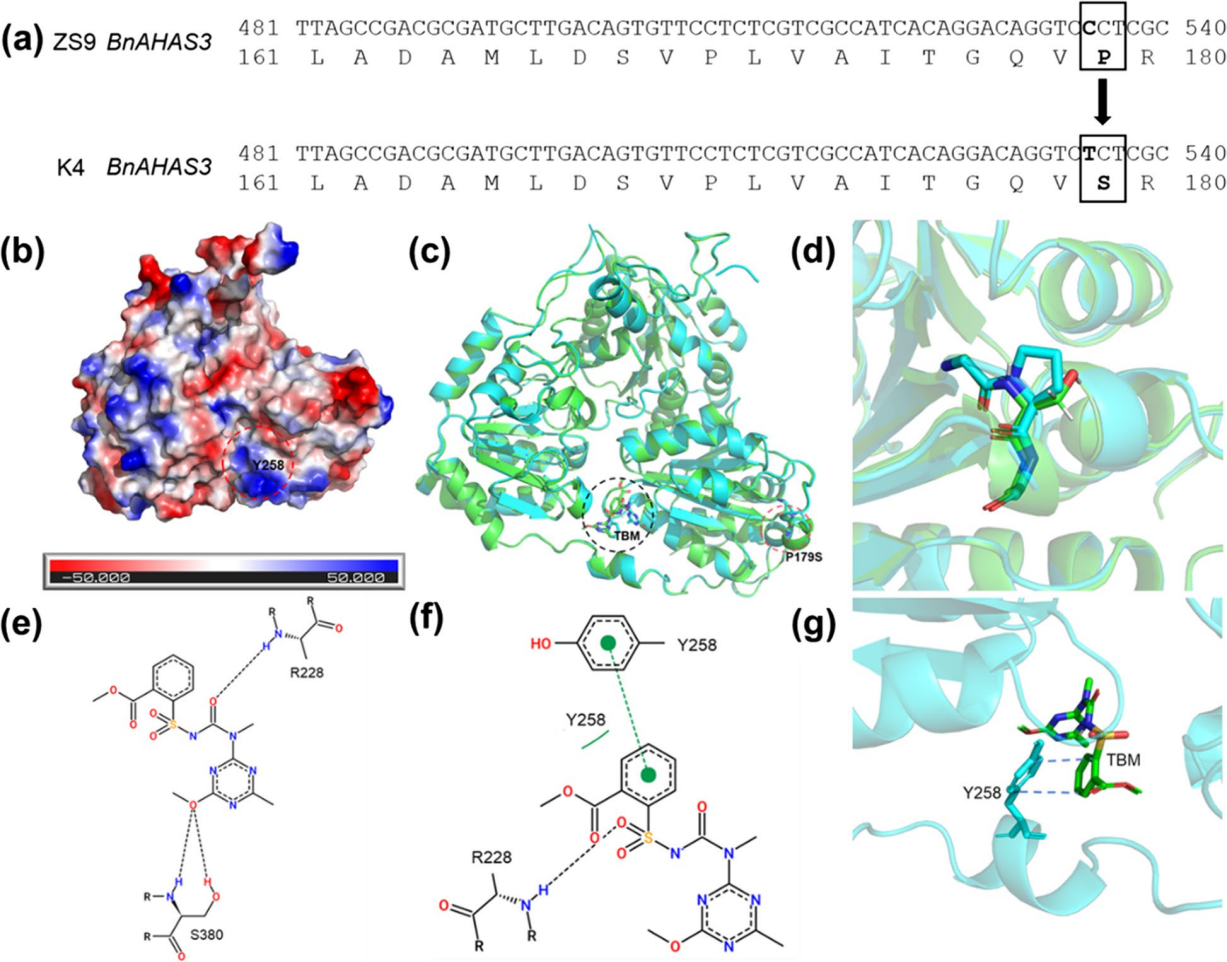
Mutant position of *BnAHAS3*^*535T*^ and three dimensional (3D) structural analysis of BnAHAS3^P179S^ in the mutant K4. **a** Partial alignment of sequence of *BnAHAS3* and its encoding protein between wild type ZS9 and the mutant K4. The numbers at right indicate the position of the last nucleotide or amino acid in the fragment illustrated. The single base substitution at 535^th^ of *BnAHAS3* coding sequence from C-T (ZS9-K4), which resulted in a substitution at point 179 (from Pro to Ser) in BnAHAS3 in the K4 (BnAHAS3^P179S^). **b** Electrostatic potential analysis of ZS9 BnAHAS3 protein surface. **c** BnAHAS3 protein after TBM treatment. **d** Comparison between the BnAHAS3 structure of ZS9 and K4 around P197S. Blue indicates ZS9 BnAHAS3 and green indicates K4 BnAHAS3^P179S^. **e** 2D interaction between BnAHAS3^P179S^ of the mutant K4 and TBM ligand. **f** 2D and (**g**) 3D diagram of the interaction between ZS9 BnAHAS3 and TBM

To figure out the critical mutation(s) in K4, three functional *BnAHAS* genes, *BnAHAS1*, *BnAHAS2*, and *BnAHAS3,* in the mutant K4 and ZS9 were amplified with the reported primers (Table S2; Hu et al. 2012). Besides, *BnAHAS3* of the other five susceptive lines (ZS7, ZS2, QSC, Q7C, and S11R) were also analyzed. Sequence analysis revealed that, compared with WT ZS9, the *BnAHAS1* and *BnAHAS2* in the K4 showed no difference. However, a single nucleotide polymorphism (SNP) at 535 (from C to T) within the coding domain of *BnAHAS3* in the K4 was detected (recorded as *BnAHAS3*^*535T*^), which lead to a substitution (from Pro to Ser) at the point 179 in BnAHAS3 (BnAHAS3^P179S^, corresponds to Pro-197 in AtAHAS) (Fig. 3a). Sequencing analysis showed that the 535th nucleotides of coding domain in *BnAHAS3* in the five susceptive lines tested were C, which was the same as that of ZS9. These results suggested that this SNP is probably responsible for the herbicide-resistance of K4 (Fig. 3a).

### Structural analysis of BnAHAS3 proteins

Hydrophobic clustering analysis was performed for BnAHAS3 in WT ZS9 and the mutant K4. The substitution of P179S in BnAHAS3 of the K4 changed the hydrophobic cluster distribution (Fig. S1a) compared with ZS9 (Fig. S1b). The hydrophilic analysis predicted that BnAHAS3 showed the highest hydrophobicity at the 352nd amino acid, with a value of 2.011, and the highest hydrophilicity at the 482nd amino acid, with a value of -2.822 (Fig. S2). The hydrophobicity of BnAHAS3^P179S^ of K4 at around of the 179th amino acid was greater than that of BnAHAS3 of ZS9 (Table S5). The results suggested that the differential response to TBM treatment might be partly caused by changes in the hydrophobic property of BnAHAS3^P179S^ in the K4. Secondary structure analysis predicted that BnAHAS3 mainly consisted of three forms of secondary structure, of which α-helix accounted for the largest proportion, however, there was no obvious difference between BnAHAS3 in ZS9 and BnAHAS3^P179S^ in the K4 (Fig. S3).

To explore the mechanism of BnAHAS3^P179S^ in response to TBM, the three-dimensional (3D) structure of BnAHAS3 and its binding with herbicide TBM were modeled. The results showed that the binding energy of BnAHAS3^P179S^ (receptor) and TBM (ligand) was -6.9 kcal.mol^−1^ in the K4, and that of BnAHAS3 and TBM was -6.6 kcal.mol^−1^ in ZS9, indicating that BnAHAS3^P179S^ in the K4 was less likely to bind to TBM. The TBM molecules contacted with BnAHAS3 near Y258, where there is a strong positive electric potential relative to the protein surface (Fig. 3b). In ZS9, the benzene ring plane of Y258 of BnAHAS3 is parallel to the benzene ring plane of TBM, forming a stable π-plane stacking effect (Fig. 3f and g). This facilitates the spatial orientation of TBM and enhances the affinity between BnAHAS3 and TBM. Thus, the catalytic function of BnAHAS3 was inhibited, which ultimately led to toxicity and even death of rapeseed plants. However, in the K4, BnAHAS3^P179S^ can alter the structure of the entire protein (Fig. 3c and d). The binding sites of TBM and BnAHAS3^P179S^ were changed accordingly, and the stable π-plane packing effect at Y258 can’t be formed, which makes BnAHAS3^P179S^ in the K4 unable to effectively bind to TBM (Fig. 3e). This ultimately leads to the K4 exhibiting tolerance to the herbicide.

### Affinity detection between TBM and BnAHAS3 by surface plasmon resonance biosensor technology

The affinity between TBM and BnAHAS3 was detected using the methodology of surface plasmon resonance (SPR). The results indicated that there were differences in the kinetic parameters of the binding process between TBM and BnAHAS3 in ZS9, and BnAHAS3^P179S^ in the K4. In the initial stage, the association rate constant *k*_a_ of BnAHAS3 in ZS9 was higher than that of BnAHAS3^P179S^ in the K4; In the equilibrium stage, the association amount between BnAHAS3 in ZS9 and TBM was significantly greater than that between BnAHAS3^P179S^ in the K4 and TBM; In the dissociation stage, the dissociation rate constant *k*_d_ of BnAHAS3 in ZS9 was lower than that of BnAHAS3^P179S^ in the K4. The affinity constant *K*_D_ of BnAHAS3 in ZS9 was lower than that of BnAHAS3^P179S^ in the K4, indicating that the affinity between BnAHAS3 in ZS9 and TBM was higher than that of BnAHAS3^P179S^ in the K4 and TBM (Table 4; Fig.S4). The R_max_ value of BnAHAS3 in ZS9 and TBM was lower than that of BnAHAS3^P179S^ in the K4 and TBM, which reflects the maximum adsorption capacity of TBM on the protein surface, however, it can’t distinguish between specific and non-specific binding, which may be due to the large amount of non-specific adsorption compared to specific binding.

**Table 4:** Dynamics parameters of the interaction between BnAHAS3 and tribenuron-methyl molecular

Sample ID	Tribenuron-methyl				
	*K*_D_ (M)	*k*_a_ (M^−1^ S^−1^)	*k*_d_ (S^−1^)	*R*_max_ (RU)	Chi^2^
BnAHAS3-ZS9	0.33 × 10^–4^	518.36	1.72 × 10^–2^	384	9.47
BnAHAS3^P179S^-K4	2.40 × 10^–4^	235.90	5.66 × 10^–2^	501	15.24

*M* mol.L^−1^, *S* second, *RU* response unit

### Ectopic expression of *BnAHAS3*^*535T*^ in *Arabidopsis* confers plant with herbicide resistance

**Fig. 4:**
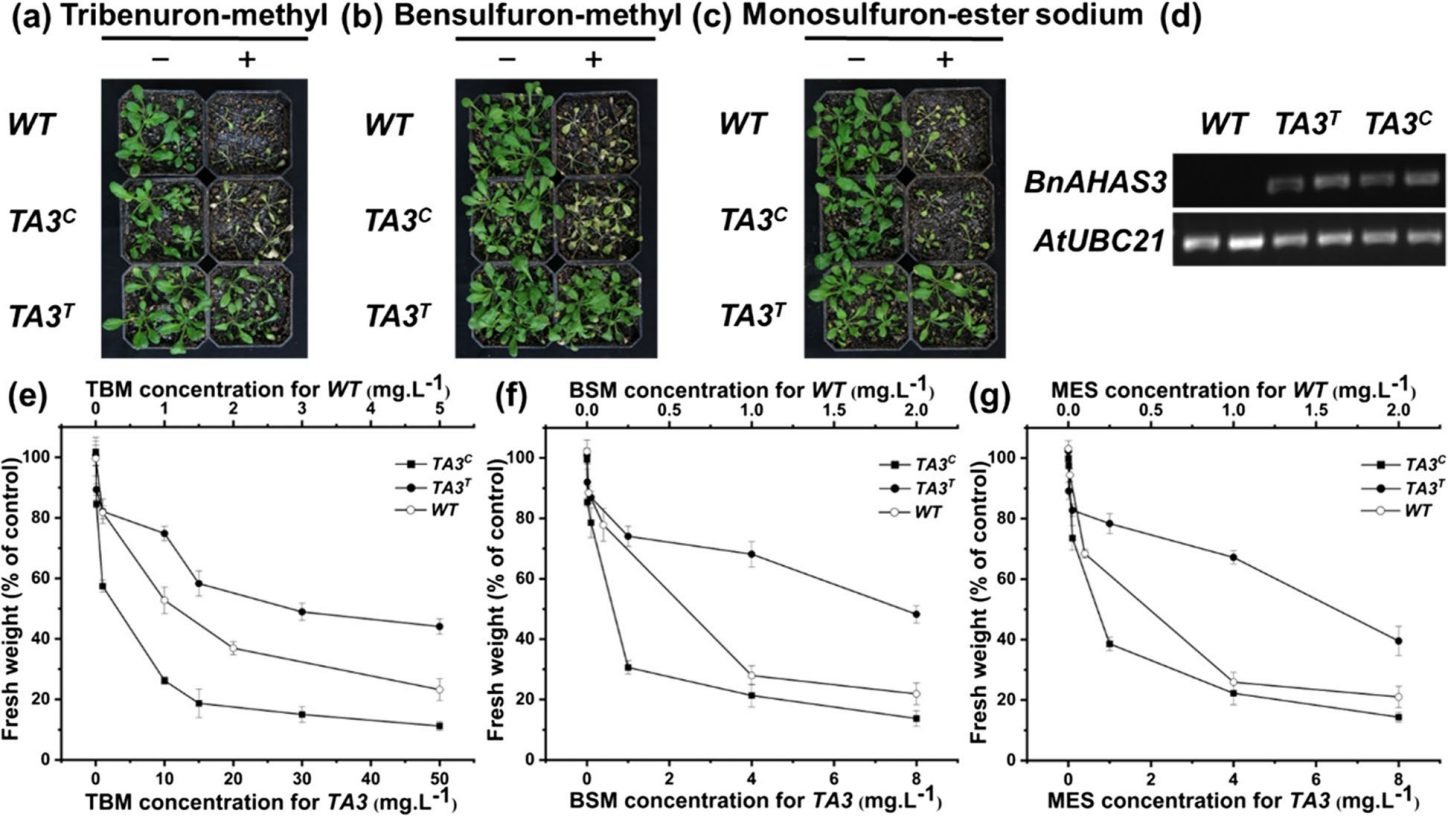
Herbicides resistance in transgenic Arabidopsis plants expressing the mutant allele *BnAHAS3*^*535T*^. Phenotype changes of Arabidopsis plants two weeks after being sprayed with 1.0 mg L^-1^ of (**a**) tribenuron-methyl; **b** bensufuron-methyl; **c** monosulfuron-ester sodium. **d** Result of SQ-PCR. *WT*, wild type Arabidopsis; *TA3*^*T*^, Arabidopsis with overexpressing the mutant allele *BnAHAS3*^*535T*^; *TA3*^*C*^, Arabidopsis with overexpressing wild type allele *BnAHAS3*^*535C*^. Fresh weight of Arabidopsis plants two weeks after being sprayed with 1.0 mg L^-1^ of (**e**) tribenuron-methyl; **f** bensufuron-methyl; **g** monosulfuron-ester sodium

In order to confirm the mutant allele *BnAHAS3*^*535T*^ contributing to herbicide-resistance in the mutant K4, a binary expression vector containing the *BnAHAS3*^*535T*^ was transformed into WT Arabidopsis, with WT allele *BnAHAS3*^*535C*^ as a control. Consequently, five independent transgenic lines with allele *BnAHAS3*^*535T*^ (TA3^T^-1 ~ TA3^T^-5) or *BnAHAS3*^*535C*^ (TA3^C^-1 ~ TA3^C^-5) were obtained through selection with 0.1% Basta. The following assays wereconducted using homozygous transgenic lines and WT Arabidopsis. Firstly, the herbicide-resistance of the transgenic lines and WT Arabidopsis was evaluated by foliar-spraying with 1.0 mg. L^−1^ of TBM, BSM, and MES, respectively. After treatment for two weeks, all the seedlings of WT Arabidopsis and transgenic lines TA3^C^-1 ~ 5 treated with the three herbicides were dead; however, the seedlings of transgenic lines TA3^T^-1 ~ 5 treated with 1.0 mg.L^−1^ of TBM and BSM showed almost no symptoms (Fig. 4a and b), while the seedlings of transgenic lines TA3^T^-1 ~ 5 treated with 1.0 mg.L^−1^ of MES suffered slight damage (Fig. 4c). Secondly, the fresh weight of transgenic lines TA3^T^-1 ~ 5, TA3^C^-1 ~ 5, and WT Arabidopsis seedlings treated with different concentrations of the three herbicides was investigated. After foliar-spraying with these herbicides for two weeks, the fresh weight of the TA3^C^-1 ~ 5 and WT seedlings treated with 0.1 mg. L^−1^ TBM dropped to 80% of those herbicide-free seedlings, however, that of the TA3^T^ seedlings required 1.0 mg L^−1^ TBM treatment to reach this level (Fig. 4e). When treated with 1.0 mg. L^−1^ BSM or MES, the fresh weight of the TA3^C^-1 ~ 5 and WT seedlings dropped to 20%-30% of the control, however, that of the TA3^T^-1 ~ 5 seedlings only dropped to 70%-80% of the control (Fig. 4f and g). Finally, by semi-quantitative PCR (SQ-PCR) analysis, *BnAHAS3*^*535T*^ and *BnAHAS3*^*535C*^ were detected in transgenic lines TA3^T^ and TA3^C^, respectively, but not in WT Arabidopsis (Fig. 4d). Together, it could be concluded that *BnAHAS3*^*535T*^ endue K4 with the ability of herbicide-resistance.

### Development of allele-specific (AS) markers and cosegregation analysis

**Fig. 5:**
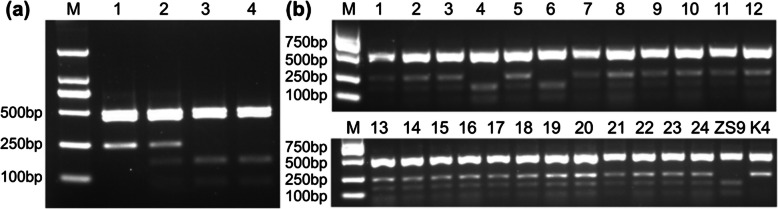
Development of co-dominant CAPS3 marker and detection of *BnAHAS3*^*535T*^ allele in F_2_ population derived from the cross between K4 and ZS9. **a** M, Marker; 1, the mutant line K4; 2, F_1_ between K4 and ZS9; 3, 4, ZS9. **b** M, Marker, No.1-No.24, F_2_ individuals derived from the cross between ZS9 and K4

Polymerase chain reaction (PCR)-based AS markers will greatly increase efficiency to select homozygous herbicide-resistant individuals with the mutant allele *BnAHAS3*^*535T*^. Therefore, several sets of AS–PCR primers were designed based on the sequence of *BnAHAS3*. One pair of the AS–PCR primers (BnAHAS3-F1 and BnAHAS3-R1) was selected (Table S1), which could only amplify the A genome-specific *BnAHAS3* in the accessions with the A genome (*B. napus*, AACC, *B. rapa*, AA) (Fig. S5). The PCR products of the lines K4 and ZS9 were approximately 1154 bp in length. Sequencing and restriction enzyme digestion site analysis indicated that PCR products of ZS9 and K4 shared two same *Ava* II restriction sites at position 459 and 692 (from the start code ATG) in *BnAHAS3*. However, in *BnAHAS3* of ZS9, the third restriction site of *Ava* II was identified at position 536, where the SNP (C/T) was located. This restriction site was used to recognize the mutant allele *BnAHAS3*^*535T*^ in K4. As a result, the amplified product of *BnAHAS3*in ZS9 could be digested into four fragments with the lengths of 485, 436, 157, and 76 bp, respectively. The agarose gel electrophoresis detection onlyshowed three bands, namely (485/436), 157, and 76 bp in length. The PCR product in the homozygous mutant (*BnAHAS3*^*535T*^) could be digested into three fragments, with lengths of 485, 436 and 233 bp, respectively. The agarose gel electrophoresis detection exhibited only two fragments with length of (485/436) and 233 bps. The PCR products in heterozygous resistant individuals (*BnAHAS3*^*535T*^/*BnAHAS3*^*535C*^) could be digested into five fragments, and the agarose gel electrophoresis detection showed four bands, with the length of (485/436), 233, 157 and 76 bps, respectively. The individual plants of ZS9, the mutant K4 and their F_1_ were detected using the AS–PCR marker, and results were obtained as expected (Fig. 5a). Moreover, the specificity of this marker was further validated by the PCR amplification and *Ava* II digestion of 24 individuals of F_2_ population derived from the cross between K4 and ZS9 (Fig. 5b).

## Discussion

Effectively controlling of weeds is one of the most important issues for rapeseed production. Utilization of herbicide-resistant rapeseed cultivars as well as the matched herbicides is the most effective and economic way for this work. Creating and characterization of rapeseed herbicide-resistant germplasm is the foundation for the development of herbicide-resistance cultivars. At present, there are abundant herbicides for controlling monocotyledonous weeds in rapeseed field, while those for controlling broadleaf (dicotyledonous) weeds are very limited (Zhang et al. 2008; Huang et al. 2020). We proposed one alternative way to effectively control weeds in rapeseed field in combination of SUs herbicides (such as TBM) with monocotyledonous herbicides, through developing rapeseed cultivars with resistance to broadleaf (dicotyledonous) herbicides (Huang et al. 2020). The single point mutation at eight loci within the highly conserved domain of AHAS has been reported, which can convert AHAS from herbicide-sensitive form to herbicide-resistant one in plants. In rapeseed, all the reported herbicide-resistant mutants were attributed to mutations at one or two of the three loci (Pro197, Trp574, and Ser653) (Hattori et al. 1995; Hu et al. 2012, 2017; Li et al. 2015; Huang et al. 2020; Guo et al. 2020, 2022). Previously, we have obtained three TBM-resistant mutants (K1, K4, and K5) derived from ZS9 via EMS mutagenesis and TBM foliar-spray screening (Qu et al. 2014; Sun et al. 2015). In the present study, the inheritance and molecular characterization of the mutant K4 was carried out. Genetic investigation indicated that the herbicide-resistance in K4 was controlled by a single dominant nuclear gene (Table 3). Molecular analysis revealed that a novel substitution of cytosine with thymine at the position 535 of *BnAHAS3* (*BnAHAS3*^*535T*^) was identified in the K4, leading to the alteration of Pro with Ser at position 179 of BnAHAS3 (BnAHAS3^P179S^, correspondence to *A. thaliana* AtAHAS-197) (Fig. 3). The mutant K4 exhibited a certain degree of cross-resistance to three herbicides TBM, BSM, and MES (Table 2). AHAS enzyme assay (Fig. 2), structural analysis of AHAS proteins (Fig. 3), SPR experiment (Table 4, Fig. S4), and the Arabidopsis transgenic experiment with *BnAHAS3*^*535T*^ (Fig. 4) confirmed *BnAHAS3*^*535T*^ to endue the K4 herbicides resistance. Although the herbicide resistance of K4 was lower than that of K5, which harbors P197S in BnAHAS1 (Huang et al. 2020), the herbicide resistant gene in K4 can be stacked with that in K5 or other resistant materials reported, using synergistic effects to develop more stronger resistant varieties (Guo et al. 2020, 2022). In addition, the mutant K4 can be used as starting material to establish novel herbicide resistant germplasm using CRISP-Cas-base editing technology (Wu et al. 2020; Cheng et al. 2021) or further EMS mutagenesis (Guo et al. 2022). Together, the mutant K4 provided a novel resource for herbicide-resistance breeding in rapeseed.

### The BnAHAS3^P179S^ in the mutant K4 provided its herbicide resistance

Major advances in the elucidation of the crystal structure of the AHAS of catalytic subunit of WT Arabidopsis or different herbicide-resistant mutants, in complex with various AHAS inhibitor herbicides have greatly improved current understanding of the detailed molecular interactions between AHAS, cofactors, and herbicides (Mccourt and Duggleby 2006; Yu and Powles 2014; Garcia et al. 2017; Lonhienne et al. 2022). The amino acid residue P197 of AHAS, which makes up the SU binding pocket, is located at the fifth-helix in the α domain of the AHAS catalytic subunit and involved in anchoring the aromatic ring through hydrophobic interactions (Mccourt and Duggleby 2006; Duggleby et al. 2008; Lonhienne et al. 2022). The substitution of P197 in AHAS to different amino acids has been reported to result in different herbicide resistance in different plant species including *A. thaliana* (Haughn and Somerville 1990), soybean (Ghio et al. 2013), peanut (Shi et al. 2023), and many weeds species (Tranel et al. 2024). In the present study, the transgenic Arabidopsis experiment using *BnAHAS3*^*535T*^ indicated that overexpression of *BnAHAS3*^*535T*^ conferred to three herbicides (TBM, BSM, and MES) resistance (Fig. 4). Hydrophobic clustering analysis (Fig. S1, Table S5) suggested that the TBM-resistance might be partly caused by changes in the hydrophobic property of BnAHAS3^P179S^ in the mutant K4. The 3D structure analysis indicated that BnAHAS3^P179S^ in the mutant K4 will alter the structure of the entire protein, which resulted in BnAHAS3^P179S^ in K4 was less likely to bind to TBM (Fig. 3). SPR results confirmed that the affinity between BnAHAS3 in ZS9 and TBM was higher than that between BnAHAS3^P179S^ in K4 and TBM (Fig.S4; Table 4). Finally, the mutant allele *BnAHAS3*^*535T*^ leads to the mutant K4 with resistance to the herbicide. Furthermore, the AS–PCR marker developed on the basis of *BnAHAS3*^*535T*^ allele, was also co-segregated with herbicide resistance (Fig. 5). Collectively, our experiments confirmed that the newly discovered BnAHAS3^P179S^ in K4 provided herbicide resistance.

### SPR technology is a promising tool to study the interaction between herbicide and its target enzyme in rapeseed

Surface Plasmon Resonance (SPR) is an optical analysis technique based on the interaction between surface plasmon resonance and evanescent waves (Mendelsohn and Brent 1999). The SPR technology is used for real-time analysis, simple and rapid monitoring of DNA–protein, protein–protein, drug-protein, nucleic acid-nucleic acid, antigen–antibody, receptor-ligand interactions among biomolecules. This technology has a wide range of applications in life sciences, medical testing, drug screening, food testing, environmental monitoring, drug testing, and forensic identification (Schuster et al. 1993; Mendelsohn and Brent 1999; Ravindran et al. 2021). To our knowledge, this is the first report to use the SPR technology to investigate the interaction between the herbicide (TBM) and target enzyme (BnAHAS3) in rapeseed. It can be seen that SPR could become a promising tool in the investigation of herbicide-resistance mechanism in other crops.

### Suitable molecular markers for the detection of the mutant allele*BnAHAS3*^*535T*^ in rapeseed

The availability of suitable molecular markers will facilitate the effective screening of herbicide-resistant materials in the rapeseed breeding program. Previously, various molecular markers have been developed to discriminate the allelic variation for *AHAS* genes. Hu et al. (2015, 2020) developed the AS–PCR and KASP (Kompetitive allele specific PCR) markers to distinguish IMI-resistant materials, which contained the BnAHAS1^S653A^ resistant allele from the mutant M9. Hu et al. (2017) developed the CAPS markers to differentiate TBM- and IMI-resistant materials, which contained the BnAHAS3^T574L^ resistant allele from the mutant M342. Li et al. (2015) developed the derived cleaved amplified polymorphic sequences (CAPS) marker to differentiate TBM-resistant materials, which contained the BnAHAS3^S197L^ allele from the mutant M45. Shi et al. (2023) developed functional KASP markers based on two SNPs for *BnAHAS1*^G1676T^ and *BnAHAS3*^G1667T^ in the resistant line 5N (Guo et al. 2020) and validated in three distinct BC_1_F_2_ populations. In the present study, one AS–PCR marker was developed on the basis of the restriction sites of the endonuclease *Ava* II, which can effectively identify homozygous and heterozygous individuals with the resistant *BnAHAS3*^*535T*^ allele (Fig. 5). The AS–PCR marker also co-segregated with the herbicide resistance in the F_2_ population derived from the cross between K4 and ZS9 (Fig. 5), suggesting that this marker can be used in future rapeseed breeding for herbicide resistance.

In a conclusion, the mutant K4 had a certain degree of resistance to three herbicides TBM, BSM, and MES. The herbicides-resistance of the mutant K4 was controlled by one dominant nuclear gene. AHAS enzyme assay, structural analysis of AHAS proteins, affinity detection between TBM and AHAS by SPR analysis, and the Arabidopsis transgenic experiment confirmed that the *BnAHAS3*^*535T*^ conferred herbicide resistance to the K4. The findings indicated that the rapeseed mutant line K4 is a value herbicide-resistance germplasm for rapeseed breeding program.

## Materials and methods

### Plant materials and herbicides

Eight rapeseed (*B. napus*) accessions K4, ZS9, ZS7, ZS2, QSC, Q7C, S11R, and SH11, two *B. rapa* (AA, 2n = 20) accessions 0B77 and 0B90, seven *B. oleracea* (CC, 2n = 18) accessions 7E108, Ribenpielan, Ganlanzijiaoxi, WanfengGanlan, Xiaguang, Jinxuan 8398, and Helan 83, were used in the present study. K4 was a TBM-resistant mutant line derived from ZS9 via EMS mutagenesis and obtained by TBM foliar-spray screening (Qu et al. 2014; Sun et al. 2015). At the end of September during years 2014–2018, seeds of all accessions were sown in the experimental field of Northwest A&F University (N34.29°, E108.06°), Yangling, Shaanxi, China. All accessions were grown in 2.0 m long rows, with 0.5 m between rows and 0.15 m within rows. Cultural practices, including soil preparation, fertilizer, and irrigation, were the same across trials. At 4–6 leaf stage, rapeseed seedlings were sprayed with different herbicides accordingly. The resistance was evaluated three weeks after herbicides treatment.

*Arabidopsis thaliana* (Col-0 ecotype) plants and its transgenic variants were grown at 22 °C under a 16 h/8 h light/dark cycle and a light intensity of 70–150 μmol m^−2^ s^−1^ and approximately 60% relative humidity in phytotron.

The four different herbicides and their rates used in the present study are shown in Table 1. Tribenuron-methyl (TBM, MaiFa®, 10% active ingredients), bensulfuron-methyl (BSM, DaoWucao®, 10% active ingredients), and florasulam (FU, FuMeiShi®, 50 g.L^−1^ active ingredients) were produced by Hetian Chemical Co. Ltd. (Shenyang, China), Kuaida Agrochemical Co. Ltd. (Jiangsu, China), and Agricultural Hormone Engineering Hormone Co. LTD (Jiangsu, China), respectively. Monosulfuron-sodium (MES, 97.5% active ingredients) was kindly provided by Professor Zhengming Li of NanKai University, Tianjin, China.

### Cross-resistance of the mutant line K4 to different herbicides

The four herbicides, including TBM, MES, FU, and BSM, were used to evaluate herbicide-resistance of rapeseed lines K4 and ZS9 at 4–6 leaf stage by foliar-spraying at different rates in a volume of 300 L ha^−1^ (Table 1). The symptoms were recorded approximately three weeks later according to Gao et al. (2010). Five indices (phytotoxicity, leaf angle, leaf numbers, fresh weight, and dry weight) were scored for each herbicide treatment.

Phytotoxicity was scored at seven grading standards described by Huang et al. (2020). Phytotoxicity index was calculated by following formula:

$$\text{Phytotoxicity index =} \sum\frac{\mathrm{score}\;\mathrm{of}\;\mathrm{the}\;\mathrm{standard}\times\mathrm{No}.\;\mathrm{of}\;\mathrm{plants}\;\mathrm{for}\;\mathrm{corresponding}\;\mathrm{standard}}{\mathrm{total}\;\mathrm{No}.\;\mathrm{of}\;\mathrm{plants}\times7}$$

The leaf angle, leaf numbers, fresh weight, and dry weight were investigated according to the method described by Xin et al. (2014).

### Enzyme extraction and assays of AHAS activity

Three weeks after herbicide treatments (Table 1), the leaves from 10 seedlings of each herbicide treatment of both rapeseed lines ZS9 and K4 were collected for AHAS enzyme activity assays according to the protocol described by Lv et al. (2018). The specific activities of AHAS of both lines were estimated by means of the zero-herbicide control. Three biological replications were conducted. AHAS enzyme extracted from the controls of both lines was also used for in vitro AHAS activity evaluation. The extracted enzyme from the controls of both lines was detected on 8% sodium dodecyl sulfate–polyacrylamide gel electrophoresis (SDS-PAGE) and stained with Coomassie Brilliant Blue R250 (Schagger and Von Jagow 1987). A mid-range protein molecular weight marker (Catalog BM524, GeneRay) was used for size estimation of enzyme. The concentrations of TBM used in in vitro assay were 0, 1, 2, 5, 10 and 20 mg L^−1^ according to the protocol of Li et al. (2015). Three biological replications were included for each assay. Data from each line were fit to a nonlinear regression model by curve fit of SigmaPlot 12.0 software (Systat Software, San Jose, CA, USA). The nonlinear regression was based on a logistic function described by Seefeldt et al. (1995):

1 $$y\;=\;c\;-\;\left(D-C\right)/\left[1\;+\;x/I_{50}\right]^b$$

Where y is the AHAS activity (% of the mean of the zero-herbicide control); x is the concentration of TBM used in the enzyme assay; C and D are the lower and upper asymptotes of AHAS activity, respectively; *I*_*50*_ is the herbicide rate required to reduce the AHAS activity by 50%; and *b* is the slope of the curve at approximately *I*_*50*_. The means of each treatment were estimated by PROC ANOVA of IBM SPSS Statistics 20.0 (IBM Corp 2013) and plotted on the logistic dose response curves.

### Genetic analysis of TBM-resistance in the mutant K4

The mutant line K4 was crossed with lines ZS9 and SH11 to develop F_1_. F_1_ plants were self-pollinated and backcrossed to the susceptible parents to obtain the F_2_, BC_1_ generations, respectively. By the end of September of the two crop seasons of 2017–2018, the seeds of all materials, including parents, F_1_, F_2_ and BC_1_ progenies, were sown in the experimental field of Northwest A&F University. The seedlings of above rapeseed materials were sprayed with 4.5 g a.i. ha^−1^ TBM in a volume of 300 L ha^−1^ at the 4–6 leaf stage. The resistance was investigated three weeks after TBM treatment. Plants were scored as resistant if they showed no significant symptom or only slight injury, and as sensitive if they died. The segregation of each population was assessed using a chi-square (χ^2^_c_) goodness-of-fit test.

### Amplification and sequence analysis of *BnAHAS1-3*

The genomic DNA of rapeseed lines ZS9, K4, ZS7, ZS2, QSC, Q7C, and S11R was extracted from 0.5 g young leaves by cetyltrimethyl ammonium bromide (CTAB) method (Doyle and Doyle 1990). *BnAHAS1*, *BnAHAS2*, and *BnAHAS3* in lines ZS9 and K4 were amplified by three pairs of primers reported by Hu et al. (2012, Table S1). Subsequently, *BnAHAS3* in lines ZS7, ZS2, QSC, Q7C, and S11R was also analyzed. The PCR protocol was the same as Huang et al. (2020). The PCR experiments were conducted triplicate for each of the three *BnAHAS* genes. The PCR products were purified from agarose gel with Gel Extraction Kit (TianGen, China), then ligated to pMD^TM^19-T Vector (Takara, China) and transformed into DH5α competent cells. Five positive clones from each PCR product of each *BnAHAS* gene were selected for sequencing by a commercial sequencing service (ShengGong, China).

The obtained sequences were analyzed using DNAMAN 6.0 (Lynnon Corporation DNAMAN-Bioinformatics Solutions, https://www.lynnon.com/downloads.html). The *AHAS* sequence of *A. thaliana* (*At3g48560*) was obtained from TAIR (The Arabidopsis Information Resource, https://www.arabidopsis.org/). Multiple sequence alignments were performed using the BioEdit 7.0 (Hall 1999).

### Arabidopsis transformation with *BnAHAS3*^*535T*^ and herbicides treatment

Sequence analysis of *BnAHAS1-3* revealed that a substitution from C to T in 535th of *BnAHAS3* coding domain was detected in the mutant line K4, and the mutant allele was named as *BnAHAS3*^*535T*^. The expression vector pCAMBIA3301 containing *BnAHAS3*^*535T*^ was constructed and transformed into *Agrobacterium tumefaciens* GV3101 as described in our previous study (Huang et al. 2020). The obtained positive clones were used to introduce *BnAHAS3*^*535T*^ into *A. thaliana* (Col-0) using floral dip transformation (Zhang et al. 2006). WT allele *BnAHAS3*^*535C*^ obtained from rapeseed line ZS9 was also used as control and transformed into *A. thaliana* (Col-0). To determine whether *BnAHAS3*^*535T*^ was the causal gene of resistance to TBM, BSM, and MES or not, at the 4–6 leaf stage, the obtained positive lines of *BnAHAS3*^*535T*^ and *BnAHAS3*^*535C*^, and WT Arabidopsis were treated with different rates of TBM, BSM, and MES solutions (Table S2).

### RNA extraction and SQ-PCR

Young leaves of homozygous *BnAHAS3*^*535T*^ and *BnAHAS3*^*535C*^ transgenic and WT Arabidopsis plants were collected for RNA isolation using plant RNA extraction kit (E.Z.N.A.®Plant RNA Kit, R6827-01, OMEGA). GoScript TM Reverse Transcription System (A5001, Promega) was utilized for the synthesis of the first strand cDNA. The SQ-PCR primers TA3-F and TA3-R (Table S1) were used to amplify the specific fragments in *BnAHAS3*. The housekeeping gene *UBC21* was employed as the reference. The primers UBC_qPCR-F and UBC_qPCR-R for *UBC21* amplification are shown in Table S1.

### Protein structure prediction

Hydrophobic cluster analysis of rapeseed BnAHAS3 proteins was performed using HCA 1.0.2 software, available at the Mobyle Portal (https://mobyle.rpbs.univ-paris-diderot.fr/cgi-bin/portal.py?form=HCA%23forms::HCA#forms::HCA). The hydrophilicity and hydrophobicity of BnAHAS3 were predicted by ProtScale in Expasy (https://web.expasy.org/protscale/). One-to-one threading was performed for secondary structure prediction by the PHYRE2 protein modeling server (http://www.sbg.bio.ic.ac.uk/phyre2/html/page.cgi?id=index_advanced.). The SWISS-MODEL web server (https://swissmodel.expasy.org/interactive/) was used for protein homology modeling to receptor protein after submission of the amino acid sequences of BnAHAS3. The 2D structure of TBM was downloaded from Pubchem website (https://pubchem.ncbi.nlm.nih.gov/) and converted into 3D structure with chemBio 3D software to obtain ligands. AutoDock Tools software was used for protein–ligand docking analysis, and the PyMOL molecular graphics system was programmed for protein structural visualization and superposition. The 2D interaction diagram of BnAHAS3 protein and TBM ligand was obtained on the Proteins plus website (https://proteins.plus/).

### Surface Plasmon Resonance (SPR) analysis

Affinity between herbicide TBM and rapeseed BnAHAS3 protein was detected by SPR technology according to the protocols described by Chen et al. (2005) and Shen et al. (2011). Briefly, total protein was isolated from rapeseed seedlings of ZS9 and K4 according to the protocol described by Lv et al. (2018), then by utilizing the differences in isoelectric point and molecular weight between different proteins, BnAHAS3 was isolated and purified from the total proteins by removing other proteins through physical or chemical methods (Karaca et al. 2011). The product was diluted to 28.4 μg mL^−1^ with acetate (pH 5.0) and the supernatant was centrifuged, select 2, 3 channels of series sensor chip CM5(Pharmacia biosensor, Sweden), the antibody coupling experiment is carried out. Then, adjust the TBM to 2000 μM with pH7.4 HBS-EP buffer (0.01 mol.L^−1^ HEPES, 0.15 mol.L^−1^ NaCl, 3 mmol.L^−1^ EDTA, and 0.005% (W/V) polysorbate 20) and centrifuge for supernatant, dilute TBM to 1000, 500, 250, 125, 62.5, 31.25, 15.62, 7.81, 3.91, 1.95, 0.98, 0.49, and 0.24 μM. Set the flow rate to 30 μL min^−1^, the combination time to 180 s, the dissociation time to 300 s, the default reaction temperature to 25 ℃, and then use the Biacore T200 (Cytova, USA) instrument for detection (Chen et al. 2005).

The kinetic parameters of the BnAHAS protein's action on TBM can be calculated using ProtenOn Manager 2.1.2 software.

$$K_D=k_d/k_a$$

Where* k*_a_, the association rate constant, *k*_d_, the dissociation rate constant, and *K*_D_, the dissociation equilibrium constant.

### Development of CAPS markers for*BnAHAS*^*3535T*^

Sequence comparison of *BnAHASs* revealed that the nucleotide sequences 531–535 bp from the translation starting site in the WT allele of *BnAHAS3* in line ZS9 was GGTCC, which was mutated to GGTC*T* in the mutant line K4. Therefore, the restriction endonuclease *Ava* II with the restriction sites GGWCC was employed to develop the CAPS marker for the detection of the causal point mutation in *BnAHAS3.* A pair of AS-PCR primers (BnAHAS3-F1 and BnAHAS3-R1, Table S1) was designed to amplify the target sequence containing the mutant locus. The PCR mixture (20 μL) contained 40 ng of template DNA, 0.40 μM each primer and 10 μL 2 × Rapid Tag MasterMix (Vazyme Biotech Co. Ltd, China). PCR amplification was performed as follows: pre-denaturation at 95 °C for 5 min; 34 cycles at 95 °C for 20 s, 58 °C for 20 s, 72 °C for 30 s; finally, extension at 72 °C for 5 min. 10 μL PCR products were digested with 3 U *Ava* II (New England Biolabs, USA) for 60 min at 37 °C at a final volume 25 μL. Subsequently, these products were separated on 2.5% (W/V) agarose gel, stained with ethidium bromide and visualized using gel imaging system (Alpha Innotech, Shanghai, China).

### Data analysis

For variance analysis of the tested traits, including the leaf angle, leaf number, fresh weight, dry weight, AHAS relative activity, and agronomic traits, a randomized complete block design was used by SPSS 19.0 software (IBM Corp 2013).

## Supplementary Information

Supplementary Material 1: Fig. S1. Analysis of partial hydrophobic clusters of amino acid sequence of BnAHAS3 in wild type ZS9 and the mutant K4. (a) Amino acid sequence of BnAHAS3 P179S of the mutant K4, (b) Amino acid sequence of BnAHAS3 of wild type ZS9. Hydrophobic amino acids are indicated in green and hydrophobic clusters are outlined in black. Red box indicates differences in the clusters due to a single amino acid change (P179S) in BnAHAS3 of the mutant K4.

Supplementary Material 2: Fig. S2. Hydrophilic/hydrophobic analysis of BnAHAS3 P179S of the mutant K4.

Supplementary Material 3: Fig. S3. Secondary structure prediction of rapeseed BnAHAS3 proteins using the PHYRE2 protein modeling server. (a) BnAHAS3 P179S of the mutant K4, (b) BnAHAS3 of wild type ZS9. Green spirals and blue arrows indicate α-helices and β-strands, respectively. Blue asterisk and red box indicate a single amino acid change (P179S) between BnAHAS3 of ZS9 and BnAHAS3 P179S of the mutant K4.

Supplementary Material 4: Fig. S4. Kinetic analysis of the interaction between BnAHAS3 and tribenuron methyl with different concentrations. (a) BnAHAS3 of ZS9; (b) BnAHAS3 P179S of K4; RU, response unit.

Supplementary Material 5: Fig. S5. PCR amplification of CAPS3 marker in 11 cultivars in three Brassica crops. M, DS2000 Marker; 1, 0B77(AA); 2, 0B90(AA); 3, 7E108(CC); 4, Ribenpielan (CC); 5, Ganlanzijiaoxi (CC); 6, WanfengGanlan (CC); 7, Xiaguang (CC); 8, Jinxuan 8398(CC); 9, Helan 83(CC); ZS9, (AACC); K4, (AACC).

Supplementary Material 6: Table S1. Primers used in the present study.

Supplementary Material 7: Table S2. Herbicides and their contents used for herbicide-resistant test for wild type Arabidopsis thaliana , transgenic BnAHAS3 *535C* and BnAHAS3 *535T* variants.

Supplementary Material 8: Table S3. Analysis of variance of five indexes of lines ZS9 and K4.

Supplementary Material 9: Table S4. Effect of foliar-spraying of three different herbicides on plant morphology of lines ZS9 and K4.

Supplementary Material 10: Table S5. Comparison of hydrophilic/hydrophobic values of BnAHAS3 of ZS9 and BnAHAS3 P179S of the mutant K4 in Brassica napus L.

## Data Availability

All data and materials are available in the paper and online supplemental files.
