# Correction: Genetic characterization of the *AHAS* mutant line K4 with resistance to AHAS-inhibitor herbicides in rapeseed (*Brassica napus* L*.*)

**DOI:** 10.1007/s44154-025-00256-3

**Published:** 2025-07-07

**Authors:** Yani Zhang, Qianxin Huang, Shengnan Wang, Lianliang Gao, Gaoping Qu, Yuan Guo, Zhaoxin Hu, Shengwu Hu

**Affiliations:** 1 College of Agronomy, Northwest A&F University, Yangling, Shaanxi 712100 China; 2 Department of Electrical and Computer Engineering, University of California San Diego, La Jolla, CA 92093 USA

**Correction** **:** **Stress Biology 5, 16 (2025)**

**https://doi.org/10.1007/s44154-024-00184-8**

Following publication of the original article (Zhang et al. 2025), The grant number of The National Natural Science Foundation of China has been updated from 3217150949 to 32172075.

The original article (Zhang et al. 2025) has been updated.
