# Supplementary material for: Genetic characterization of the AHAS mutant line K4 with resistance to AHAS-inhibitor herbicides in rapeseed (Brassica napus L.)
Source: Stress Biol. 2025 Feb 25;5(1):16. doi: 10.1007/s44154-024-00184-8 (PMC11861483; doi:10.1007/s44154-024-00184-8)
Supplement: Supplementary file 1 — Supplementary Material 1: Fig. S1. Analysis of partial hydrophobic clusters of amino acid sequence of BnAHAS3 in wild type ZS9 and the mutant K4. (a) Amino acid sequence of BnAHAS3 P179S of the mutant K4, (b) Amino acid sequence of BnAHAS3 of wild type ZS9. Hydrophobic amino acids are indicated in green and hydrophobic clusters are outlined in black. Red box indicates differences in the clusters due to a single amino acid change (P179S) in BnAHAS3 of the mutant K4. [file 44154_2024_184_MOESM1_ESM.docx]

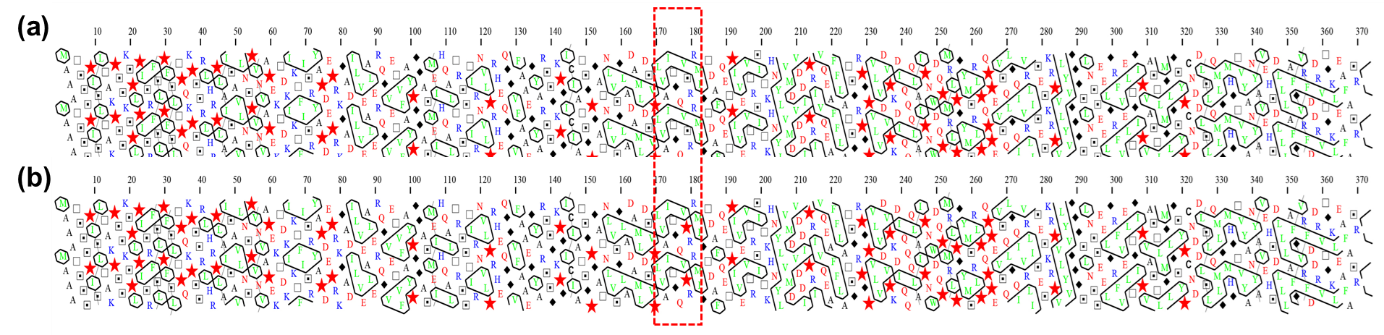
**Fig. S1** Analysis of partial hydrophobic clusters of amino acid sequence of AHAS3 in wild type ZS9 and the mutant K4. (a) Amino acid sequence of AHAS3 of the mutant K4, (b) Amino acid sequence of AHAS3 of wild type ZS9. Hydrophobic amino acids are indicated in green and hydrophobic clusters are outlined in black. Red box indicates differences in the clusters due to a single amino acid change between wild type ZS9 and the mutant K4.
