# Supplementary figures and images for: Genetic characterization of the AHAS mutant line K4 with resistance to AHAS-inhibitor herbicides in rapeseed (Brassica napus L.)
Source: Stress Biol. 2025 Feb 25;5(1):16. doi: 10.1007/s44154-024-00184-8 (PMC11861483; doi:10.1007/s44154-024-00184-8)

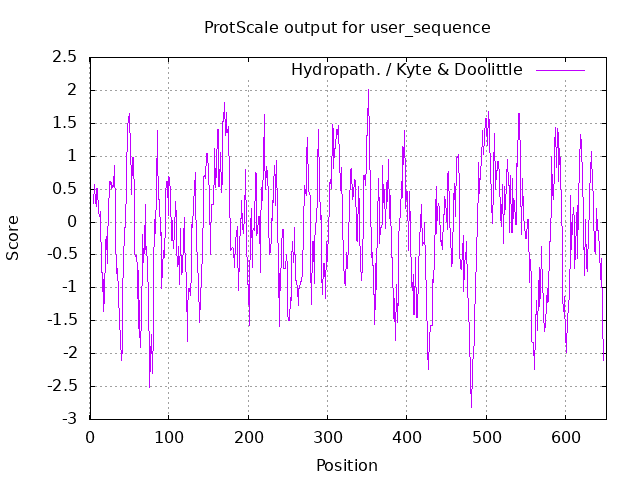
**Fig. S2** Hydrophilic/hydrophobic analysis of *Bn*AHAS3 of the mutant K4

Supplement: Supplementary file 2 — Supplementary Material 2: Fig. S2. Hydrophilic/hydrophobic analysis of BnAHAS3 P179S of the mutant K4. [file 44154_2024_184_MOESM2_ESM.docx]
