# Supplementary material for: Genetic characterization of the AHAS mutant line K4 with resistance to AHAS-inhibitor herbicides in rapeseed (Brassica napus L.)
Source: Stress Biol. 2025 Feb 25;5(1):16. doi: 10.1007/s44154-024-00184-8 (PMC11861483; doi:10.1007/s44154-024-00184-8)
Supplement: Supplementary file 3 — Supplementary Material 3: Fig. S3. Secondary structure prediction of rapeseed BnAHAS3 proteins using the PHYRE2 protein modeling server. (a) BnAHAS3 P179S of the mutant K4, (b) BnAHAS3 of wild type ZS9. Green spirals and blue arrows indicate α-helices and β-strands, respectively. Blue asterisk and red box indicate a single amino acid change (P179S) between BnAHAS3 of ZS9 and BnAHAS3 P179S of the mutant K4. [file 44154_2024_184_MOESM3_ESM.docx]

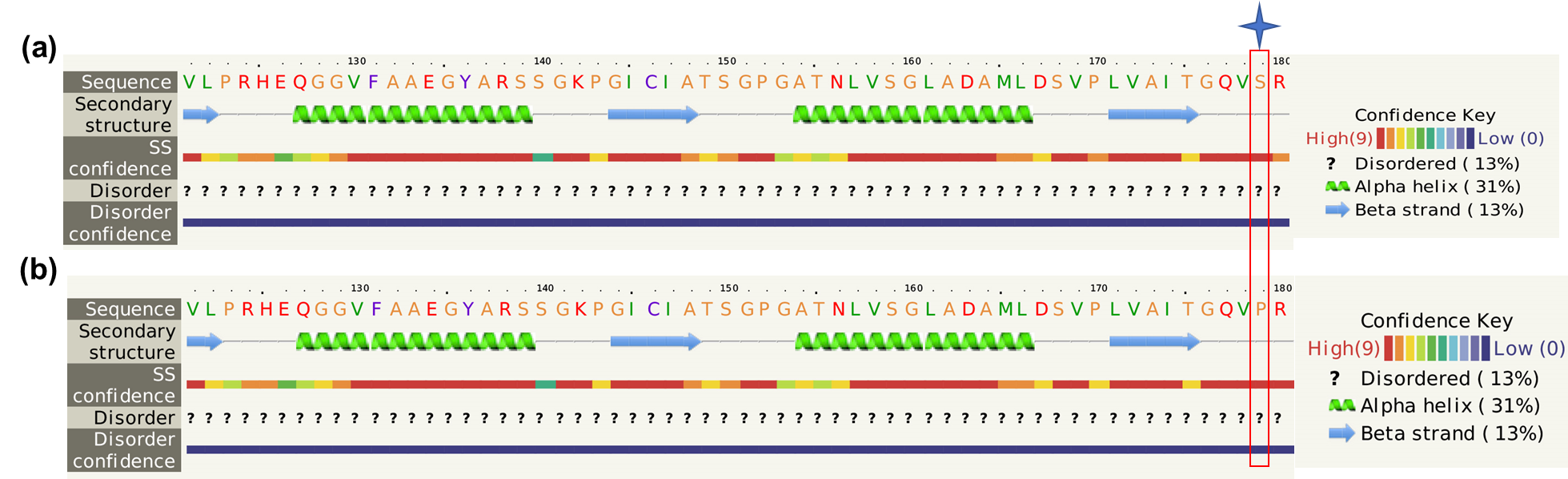
**Fig. S3** Secondary structure prediction of rapeseed *Bn*AHAS3 proteins using the PHYRE2 protein modeling server. (a) *Bn*AHAS3 of the mutant K4, (b) *Bn*AHAS3 of wild type ZS9. Green spirals and blue arrows indicate α-helices and β-strands, respectively. Blue asterisk and red box indicate mutant base substitution changes in *Bn*AHAS3 proteins.
