# Supplementary material for: Genetic characterization of the AHAS mutant line K4 with resistance to AHAS-inhibitor herbicides in rapeseed (Brassica napus L.)
Source: Stress Biol. 2025 Feb 25;5(1):16. doi: 10.1007/s44154-024-00184-8 (PMC11861483; doi:10.1007/s44154-024-00184-8)
Supplement: Supplementary file 4 — Supplementary Material 4: Fig. S4. Kinetic analysis of the interaction between BnAHAS3 and tribenuron methyl with different concentrations. (a) BnAHAS3 of ZS9; (b) BnAHAS3 P179S of K4; RU, response unit. [file 44154_2024_184_MOESM4_ESM.docx]

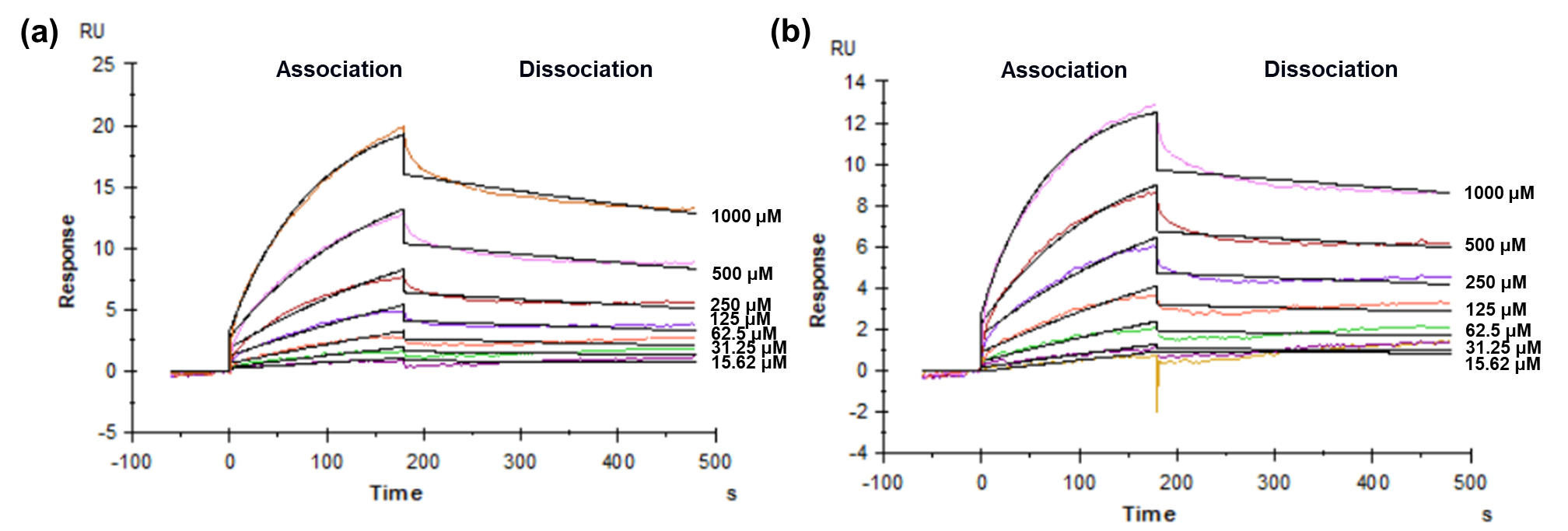
**Fig. S4** Kinetic analysis of the interaction between *Bn*AHAS protein and tribenuron methyl with different concentrations. (a) *Bn*AHAS3-ZS9; (b) *Bn*AHAS3-K4; RU, response unit.
