# Supplementary material for: Genetic characterization of the AHAS mutant line K4 with resistance to AHAS-inhibitor herbicides in rapeseed (Brassica napus L.)
Source: Stress Biol. 2025 Feb 25;5(1):16. doi: 10.1007/s44154-024-00184-8 (PMC11861483; doi:10.1007/s44154-024-00184-8)
Supplement: Supplementary file 5 — Supplementary Material 5: Fig. S5. PCR amplification of CAPS3 marker in 11 cultivars in three Brassica crops. M, DS2000 Marker; 1, 0B77(AA); 2, 0B90(AA); 3, 7E108(CC); 4, Ribenpielan (CC); 5, Ganlanzijiaoxi (CC); 6, WanfengGanlan (CC); 7, Xiaguang (CC); 8, Jinxuan 8398(CC); 9, Helan 83(CC); ZS9, (AACC); K4, (AACC). [file 44154_2024_184_MOESM5_ESM.docx]

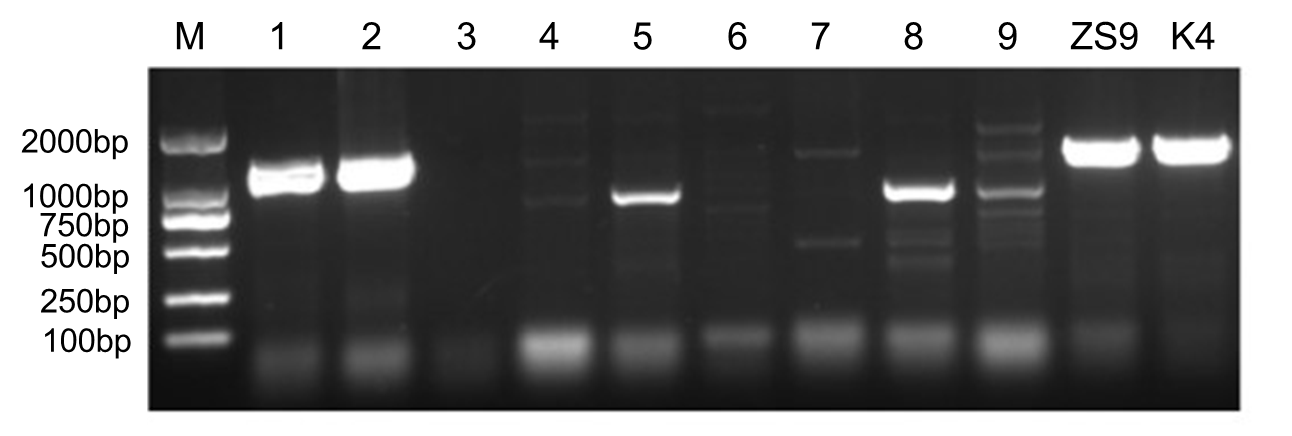
**Fig. S5** PCR amplification of CAPS3 marker in 11 cultivars in three Brassica crops.

M, DS2000 Marker; 1, 0B77 (AA); 2, 0B90 (AA); 3, 7E108 (CC); 4, Ribenpielan (CC); 5, Ganlanzijiaoxi (CC); 6, WanfengGanlan (CC); 7, Xiaguang (CC); 8, Jinxuan 8398 (CC); 9, Helan 83 (CC); ZS9, (AACC); K4, (AACC).
