# Supplementary material for: Genetic characterization of the AHAS mutant line K4 with resistance to AHAS-inhibitor herbicides in rapeseed (Brassica napus L.)
Source: Stress Biol. 2025 Feb 25;5(1):16. doi: 10.1007/s44154-024-00184-8 (PMC11861483; doi:10.1007/s44154-024-00184-8)
Supplement: Supplementary file 6 — Supplementary Material 6: Table S1. Primers used in the present study. [file 44154_2024_184_MOESM6_ESM.docx]

**Table S1** Primers used in the present study

| Primer name | Sequence (5’- 3’) | Purpose | The reference gene | Reference |
| --- | --- | --- | --- | --- |
| BnAHAS3-F | CTCTCTCTCTCTCATCTAACCAT | *BnAHAS3* amplification |  | Hu et al. (2012) |
| BnAHAS3-R | ACTGAAACTAAGTCTTTTACCAT |  |  |  |
| BnAHAS1-F | TCAAGAACAGTTAGATCCAC | *BnAHAS1* amplification |  |  |
| BnAHAS1-R | GATCACCAGCTTCATCTCT |  |  |  |
| BnAHAS2-F | AAGCAATTTCTCGCAACACTC | *BnAHAS2* amplification |  |  |
| BnAHAS2-R | CAGAAGAGAGCATAGAATAATCAA |  |  |  |
| BnAHAS3-F1 | CTCTCATTTCTCTCTCTCTCTCATC | Allele-specific PCR marker | Z11526 |  |
| BnAHAS3-R1 | TGCACAATCTTAGCCCTGCTCGCA |  |  |  |
| ahas3NcoI-F | CATGCCATGGATGGCGGCGGCAACATCGTCTTCTC | Arabidopsis transformation |  |  |
| ahas3NcoI-R | GCTTGGTGGATCTCCATAGAAGCACCTCCGGG |  |  |  |
| ahas3Blunt-F | CCCGGAGGTGCTTCTATGGAGATCCACCAAGC |  |  |  |
| ahas3Blunt-R | TCAGTACTTAGTGCGACCATCCCCTTC |  |  |  |
| TA3-F | ACGCCAATCGTTGAGGTA | Semi-quantitative PCR |  |  |
| TA3-R | GCAAGCTGCTGCTGAATAT |  |  |  |
| UBC_qPCR-F | CTGCGACTCAGGGAATCTTCTAA |  |  |  |
| UBC_qPCR-R | TTGTGCCATTGAATTGAACCC |  |  |  |
