# Supplementary material for: Genetic characterization of the AHAS mutant line K4 with resistance to AHAS-inhibitor herbicides in rapeseed (Brassica napus L.)
Source: Stress Biol. 2025 Feb 25;5(1):16. doi: 10.1007/s44154-024-00184-8 (PMC11861483; doi:10.1007/s44154-024-00184-8)
Supplement: Supplementary file 7 — Supplementary Material 7: Table S2. Herbicides and their contents used for herbicide-resistant test for wild type Arabidopsis thaliana , transgenic BnAHAS3 535C and BnAHAS3 535T variants. [file 44154_2024_184_MOESM7_ESM.docx]

**Table S2** Herbicides and their contents used for herbicide-resistant test for wild type *Arabidopsis thaliana*, transgenic *BnAHAS3^535C^* and *BnAHAS3^535T^* variants

| Herbicides | Contents for WT and transgenic *BnAHAS3^535C^* variants  (mg. L^-1^) | Contents for transgenic *BnAHAS3^535T^* variants  (mg. L^-1^) |
| --- | --- | --- |
| Tribenuron-methyl | 0 | 0 |
|  | 0.001 | 0.0003 |
|  | 0.01 | 0.003 |
|  | 0.1 | 0.03 |
|  | 1 | 0.3 |
|  | 2 | 0.6 |
|  | - | 5 |
|  | - | 10 |
|  | - | 15 |
|  | - | 30 |
|  | - | 50 |
| Bensulfuron-methyl | 0 | 0 |
|  | 0.001 | 0.001 |
|  | 0.01 | 0.01 |
|  | 0.1 | 0.1 |
|  | 1 | 1 |
|  | 2 | 2 |
|  | - | 4 |
|  | - | 8 |
| Monosulfon- sodium | 0 | 0 |
|  | 0.001 | 0.001 |
|  | 0.01 | 0.01 |
|  | 0.1 | 0.1 |
|  | 1 | 1 |
|  | 2 | 2 |
|  | - | 4 |
|  | - | 8 |

-, no data
