# Supplementary material for: Genetic characterization of the AHAS mutant line K4 with resistance to AHAS-inhibitor herbicides in rapeseed (Brassica napus L.)
Source: Stress Biol. 2025 Feb 25;5(1):16. doi: 10.1007/s44154-024-00184-8 (PMC11861483; doi:10.1007/s44154-024-00184-8)
Supplement: Supplementary file 8 — Supplementary Material 8: Table S3. Analysis of variance of five indexes of lines ZS9 and K4. [file 44154_2024_184_MOESM8_ESM.docx]

**Table S3** Analysis of variance of five indexes of lines ZS9 and K4

| Lines | Herbicides | Index | DF_1_ | MS_1_  (between groups) | MS_2_  (within groups) | F | *P* |
| --- | --- | --- | --- | --- | --- | --- | --- |
| ZS9 | TBM | phytotoxicity index | 6 | 0.2770 | 0 | 1018.571 | 0 |
|  |  | leaf angle | 6 | 852.180 | 4.638 | 183.723 | 0 |
|  |  | leaf number | 6 | 8.913 | 0.086 | 103.264 | 0 |
|  |  | fresh weight | 6 | 270.933 | 8.505 | 31.856 | 0 |
|  |  | dry weight | 6 | 3.146 | 0.166 | 18.955 | 0 |
| K4 | TBM | phytotoxicity index | 6 | 0.104 | 0.012 | 8.400 | 0.001 |
|  |  | leaf angle | 6 | 460.259 | 19.710 | 23.352 | 0 |
|  |  | leaf number | 6 | 5.395 | 0.283 | 19.081 | 0 |
|  |  | fresh weight | 6 | 6.343 | 1.531 | 4.143 | 0.009 |
|  |  | dry weight | 6 | 0.085 | 0.056 | 1.506 | 0.229 |
| ZS9 | BSM | phytotoxicity index | 6 | 0.196 | 0 | 531.86 | 0 |
|  |  | leaf angle | 6 | 402.381 | 14.906 | 26.995 | 0 |
|  |  | leaf number | 6 | 5.603 | 0.247 | 22.683 | 0 |
|  |  | fresh weight | 6 | 136.861 | 11.518 | 11.882 | 0 |
|  |  | dry weight | 6 | 1.390 | 0.153 | 9.113 | 0 |
| K4 | BSM | phytotoxicity index | 6 | 0.041 | 0.002 | 16.822 | 0 |
|  |  | leaf angle | 6 | 110.052 | 33.158 | 3.319 | 0.03 |
|  |  | leaf number | 6 | 1.388 | 0.435 | 3.194 | 0.034 |
|  |  | fresh weight | 6 | 40.822 | 4.341 | 9.418 | 0 |
|  |  | dry weight | 6 | 0.453 | 0.185 | 2.455 | 0.059 |
| ZS9 | MES | phytotoxicity index | 7 | 0.354 | 0 | 1341.475 | 0 |
|  |  | leaf angle | 7 | 1203.921 | 19.049 | 63.202 | 0 |
|  |  | leaf number | 7 | 6.113 | 0.169 | 36.114 | 0 |
|  |  | fresh weight | 7 | 205.682 | 5.965 | 34.481 | 0 |
|  |  | dry weight | 7 | 2.562 | 0.095 | 27.077 | 0 |
| K4 | MES | phytotoxicity index | 6 | 0.065 | 0.003 | 19.122 | 0 |
|  |  | leaf angle | 6 | 169.533 | 21.369 | 7.934 | 0.001 |
|  |  | leaf number | 6 | 2.291 | 0.167 | 13.744 | 0 |
|  |  | fresh weight | 6 | 23.74 | 7.041 | 3.371 | 0.018 |
|  |  | dry weight | 6 | 0.221 | 0.124 | 1.790 | 0.15 |

TBM, tribenuron-methyl; BSM, bensufuron-methyl; MES, monosulfuron-ester sodium; No., number; IR, inhibition rate. ZS9, Zhongshuang No.9; K4, the mutant line.
