# Supplementary material for: Genetic characterization of the AHAS mutant line K4 with resistance to AHAS-inhibitor herbicides in rapeseed (Brassica napus L.)
Source: Stress Biol. 2025 Feb 25;5(1):16. doi: 10.1007/s44154-024-00184-8 (PMC11861483; doi:10.1007/s44154-024-00184-8)
Supplement: Supplementary file 9 — Supplementary Material 9: Table S4. Effect of foliar-spraying of three different herbicides on plant morphology of lines ZS9 and K4. [file 44154_2024_184_MOESM9_ESM.docx]

**Table S4** Effect of foliar-spraying of three different herbicides on plant morphology of rapeseed lines ZS9 and K4

| Herbicides | | Lines | Herbicide rates  (g a.i. ha^-1^) | Phytotoxicity index | Leaf Angle | | Leaf number | | Fresh weight | | Dry weight | |
| --- | --- | --- | --- | --- | --- | --- | --- | --- | --- | --- | --- | --- |
|  |  |  |  |  | Angle (°) | IR (%) | No. | IR (%) | Weight (g) | IR (%) | Weight (g) | IR (%) |
| tribenuron-methyl | ZS9 | | 0 | 0.1 | 57.5 ± 11.1^a^ | 0 | 6.3 ± 1.2^a^ | 0 | 61.1 ± 15.0^a^ | 0 | 7.2 ± 2.4^a^ | 0 |
|  |  |  | 0.06 | 0.1 | 58.0 ± 16.8^a^ | 0 | 7.1±0.8a | 0 | 69.5 ± 3.9^a^ | 0 | 7.9 ± 0.4 | 10.0 |
|  |  |  | 0.15 | 0.3 | 46.0 ± 10.4^b^ | 20.0 | 5.2 ± 0.7^a^ | 20.0 | 43.2 ± 12.3^b^ | 30.0 | 5.3 ± 1.0^b^ | 30.0 |
|  |  |  | 0.3 | 0.5 | 48.5 ± 11.8^b^ | 20.0 | 5.1 ± 1.4^a^ | 20.0 | 49.2 ± 8.3^ab^ | 20.0 | 4.7 ± 1.6^b^ | 40.0 |
|  |  |  | 0.6 | 0.6 | 32.5 ± 13.3^c^ | 40.0 | 3.7 ± 0.5^b^ | 40.0 | 18.0 ± 5.0^c^ | 70.0 | 2.4 ± 0.6^c^ | 70.0 |
|  |  |  | 1.5 | 0.6 | 31.0 ± 12.0^c^ | 50.0 | 2.8 ± 0.7^c^ | 50.0 | 12.7 ± 5.0^c^ | 80.0 | 1.7 ± 0.7^c^ | 80.0 |
|  |  |  | 3.0 | 0.9 | 11.3 ± 6.9^d^ | 80.0 | 2.5 ± 0.5^c^ | 60.0 | 7.4 ± 4.3^c^ | 90.0 | 1.3 ± 0.5^c^ | 80.0 |
|  | K4 | | 0 | 0 | 50.5 ± 14.3^ab^ | 0 | 7.2 ± 0.8^ab^ | 0 | 25.7 ± 4.4^a^ | 0 | 4.6 ± 1.4^a^ | 0.0 |
|  |  |  | 1.5 | 0.1 | 59.3 ± 9.9^a^ | 0 | 7.7 ± 1.2^a^ | 0 | 25.8 ± 1.3^a^ | 0 | 4.5 ± 0.7^ab^ | 3.0 |
|  |  |  | 3.0 | 0.3 | 54.0 ± 10.9^a^ | 0 | 7.8 ± 0.9^a^ | 0 | 25.7 ± 3.3^a^ | 0 | 4.5 ± 0.7^ab^ | 3.0 |
|  |  |  | 6.0 | 0.4 | 43.0 ± 13.8^b^ | 10.0 | 6.4 ± 1.2^bc^ | 10.0 | 24.8 ± 4.6^a^ | 3.0 | 4.2 ± 0.8^ab^ | 10.0 |
|  |  |  | 15.0 | 0.4 | 42.0 ± 15.7^b^ | 20.0 | 5.9 ± 1.1^c^ | 20.0 | 21.0 ±2.4^ab^ | 20.0 | 3.7 ± 0.4^abc^ | 20.0 |
|  |  |  | 30.0 | 0.5 | 30.0 ± 15.5^c^ | 40.0 | 4.7 ± 0.8^d^ | 30.0 | 19.7 ± 3.5^ab^ | 20.0 | 3.1 ± 0.1^bc^ | 30.0 |
|  |  |  | 60.0 | 0.6 | 26.0 ± 12.8^c^ | 50.0 | 4.5 ± 0.5^d^ | 40.0 | 15.6 ± 5.7^b^ | 40.0 | 2.7 ± 1.0^c^ | 40.0 |
| bensufuron-methyl | ZS9 | | 0 | 0.1 | 50.0 ± 9.2^a^ | 0 | 5.5 ± 0.9^a^ | 0 | 66.9 ± 20.8^a^ | 0 | 7.0 ± 2.3^a^ | 0 |
|  |  |  | 0.15 | 0.1 | 44.5 ± 21.6^a^ | 10.0 | 5.8 ± 0.9^a^ | 10.0 | 38.5 ± 3.2^b^ | 40.0 | 4.8±0.7^ab^ | 30.0 |
|  |  |  | 0.3 | 0.2 | 38.0 ± 15.5^ab^ | 20.0 | 5.5 ± 1.1^a^ | 20.0 | 36.0 ± 2.9^b^ | 40.0 | 4.5±0.7^b^ | 40.0 |
|  |  |  | 0.6 | 0.3 | 42.8 ± 17.3^a^ | 20.0 | 5.7 ± 1.1^a^ | 20.0 | 34.3 ± 4.4^b^ | 40.0 | 4.1±0.6^b^ | 40.0 |
|  |  |  | 1.5 | 0.6 | 39.0 ± 20.7^ab^ | 20.0 | 2.9 ± 0.7^c^ | 60.0 | 13.5 ± 7.42^c^ | 80.0 | 1.69±0.9^c^ | 80.0 |
|  |  |  | 3.0 | 0.6 | 33.5 ± 14.6^b^ | 30.0 | 3.6 ± 0.7^b^ | 50.0 | 25.4 ± 11.2^b^ | 60.0 | 3.2 ± 1.2^b^ | 60.0 |
|  |  |  | 4.5 | 0.6 | 17.0 ± 11.1^c^ | 70.0 | 2.8 ± 0.8^c^ | 60.0 | 18.1 ± 7.6^b^ | 70.0 | 2.2 ± 0.7^b^ | 70.0 |
|  | K4 | | 0 | 0.0 | 50.5 ± 14.3^a^ | 0 | 7.2 ± 0.8^a^ | 0 | 40.1 ± 4.9^a^ | 0 | 4.6 ± 1.4^ab^ | 0 |
|  |  |  | 1.5 | 0.1 | 53.0 ± 15.5^a^ | 0 | 7.5 ± 0.9^a^ | 0 | 46.3 ± 6.9^a^ | 0 | 5.5 ± 0.6^a^ | 0 |
|  |  |  | 3.0 | 0.0 | 51.0 ± 12.0^a^ | 0 | 7.3 ± 1.2^a^ | 0 | 38.3 ± 2.9^a^ | 5.0 | 4.6 ± 0.4^ab^ | 0 |
|  |  |  | 4.5 | 0.1 | 52.5 ± 10.6^a^ | 0 | 8.0 ± 1.6^a^ | 0 | 39.3 ± 5.4^a^ | 2.0 | 4.8 ± 0.8^a^ | 0 |
|  |  |  | 9.0 | 0.2 | 43.0 ± 16.4^a^ | 15.0 | 6.7 ± 0.8^a^ | 7.0 | 24.8 ± 1.9^bc^ | 40.0 | 3.4 ± 0.3^b^ | 25.0 |
|  |  | | 13.5 | 0.4 | 36.0 ± 10.4^b^ | 30.0 | 6.7 ± 1.7^a^ | 6.0 | 27.7 ± 6.9^bc^ | 30.0 | 3.5 ± 0.8^bc^ | 25.0 |
|  |  | | 18.0 | 0.3 | 47.0 ± 10.8^a^ | 10.0 | 5.9 ± 0.7^b^ | 20.0 | 20.8 ± 2.8^c^ | 50.0 | 2.5 ± 0.4^c^ | 50.0 |
| monosulfuron-sodium | ZS9 | | 0 | 0.1 | 62.0 ± 11.5a | 0.0 | 5.3 ± 0.6a | 0 | 54.7 ± 16.7^a^ | 0 | 6.6 ± 2.2^a^ | 0 |
|  |  |  | 0.1 | 0.1 | 61.0 ± 13.9^a^ | 1.0 | 5.5 ± 0.9^a^ | 20.0 | 54.6 ± 2.4^a^ | 10.0 | 6.4 ±0.8^a^ | 10.0 |
|  |  |  | 0.2 | 0.1 | 59.5 ± 14.2^a^ | 4.0 | 4.8 ± 0.9^b^ | 30.0 | 40.9 ± 6.9^a^ | 30.0 | 4.8 ± 0.5^a^ | 30.0 |
|  |  |  | 0.5 | 0.2 | 53.5 ± 12.9^ab^ | 10.0 | 4.8 ± 0.8^b^ | 30.0 | 36.2 ± 6.3^a^ | 40.0 | 4.3 ± 0.4^a^ | 40.0 |
|  |  |  | 1.0 | 0.7 | 31.0 ± 13.3^b^ | 50.0 | 2.4 ± 0.5^b^ | 50.0 | 9.4 ± 3.5^b^ | 80.0 | 1.4 ± 0.5^b^ | 80.0 |
|  |  |  | 2.0 | 0.7 | 20.0 ± 8.6^c^ | 70.0 | 2.6 ± 0.7^b^ | 50.0 | 9.7 ± 2.5^b^ | 80.0 | 1.5 ± 0.3^b^ | 80.0 |
|  |  |  | 5.0 | 0.8 | 18.5 ± 7.5^c^ | 70.0 | 2.5 ± 0.7^b^ | 50.0 | 7.0 ± 2.0^b^ | 90.0 | 1.1 ± 0.3^b^ | 80.0 |
|  |  |  | 10.0 | 0.8 | 21.0 ± 11.6^c^ | 70.0 | 2.3 ± 0.5^b^ | 60.0 | 6.4 ± 0.8^b^ | 90.0 | 1.1 ± 0.3^b^ | 80.0 |
|  | K4 | | 0 | 0.1 | 50.5 ± 14.3^a^ | 0 | 7.2 ± 0.8^a^ | 0 | 35.7 ± 10.1^a^ | 0 | 4.6 ± 1.4^a^ | 0 |
|  |  |  | 0.5 | 0.1 | 55.0 ± 17.0^a^ | 0 | 6.3 ± 1.4^a^ | 10.0 | 37.1 ± 5.2^a^ | 8.0 | 4.2 ± 0.6^a^ | 10.0 |
|  |  |  | 1.0 | 0.2 | 54.4 ± 13.2^a^ | 0 | 6.3 ± 0.6^a^ | 10.0 | 35.2 ± 4.8^a^ | 10.0 | 3.8 ± 0.4^a^ | 20.0 |
|  |  |  | 2.0 | 0.3 | 47.5 ± 10.1^ab^ | 6.0 | 6.5 ± 1.0^a^ | 9.0 | 27.9 ± 10.2^a^ | 20.0 | 3.5 ± 1.2^a^ | 25.0 |
|  |  |  | 5.0 | 0.4 | 44.0 ± 11.8^ab^ | 10.0 | 5.5 ± 1.0^b^ | 20.0 | 22.9 ± 10.5^a^ | 35.0 | 2.9 ± 1.3^a^ | 40.0 |
|  |  |  | 10.0 | 0.4 | 40.5 ± 12.3^bc^ | 20.0 | 5.6 ± 0.9^b^ | 20.0 | 24.7 ± 4.9^a^ | 30.0 | 3.1 ± 0.7^a^ | 35.0 |
|  |  | | 20.0 | 0.5 | 35.0 ± 11.0^c^ | 30.0 | 4.8 ± 0.8^b^ | 30.0 | 20.7 ± 9.5^a^ | 40.0 | 2.7 ± 1.2^a^ | 40.0 |

Phytotoxicity is scored according to seven grading standards three weeks after spraying herbicides. 0, all leaves are green; 1, young leaves (the first and second ones) are light yellow-green; 2, Partial of young leaves are yellow; 3, the second leaf is yellow and curled; 4, the mature leaves are yellow-green or light purple; 5, some mature leaves dead; 6, the plant dead. Phytotoxicity index is calculated by the following formula:

Phytotoxicity index =$\sum\frac{\text{score of the standard × No. of plants for the corresponding standard}}{\text{total number of plants × 7}}$

Data are expressed by mean ± SD, three replications, and inhibition rate (IR) are calculated by the formula:

IR=$(1-\frac{\text{data of treatments}}{\text{data of control}} )\times100\%$

Data followed by different lower case letters within the same column indicated a significant difference at 0.05 level; g. a.i. ha^-1^, gram active ingredients hectare^-1^.
