# Supplementary material for: Genetic characterization of the AHAS mutant line K4 with resistance to AHAS-inhibitor herbicides in rapeseed (Brassica napus L.)
Source: Stress Biol. 2025 Feb 25;5(1):16. doi: 10.1007/s44154-024-00184-8 (PMC11861483; doi:10.1007/s44154-024-00184-8)
Supplement: Supplementary file 10 — Supplementary Material 10: Table S5. Comparison of hydrophilic/hydrophobic values of BnAHAS3 of ZS9 and BnAHAS3 P179S of the mutant K4 in Brassica napus L. [file 44154_2024_184_MOESM10_ESM.docx]

**Table S5** Comparison of hydrophilic/hydrophobic values of BnAHAS3 of ZS9 and BnAHAS3^P179S^ of the mutant K4 in *Brassica napus* L.

| Position | 175 | 176 | 177 | 178 | 179 | 180 | 181 | 182 | 183 |
| --- | --- | --- | --- | --- | --- | --- | --- | --- | --- |
| BnAHAS3^P179S^ Score | 1.456 | 0.533 | -0.433 | -0.422 | -0.422 | -0.389 | -0.422 | -0.422 | -0.689 |
| BnAHAS3 Score | 1.367 | 0.444 | -0.522 | -0.511 | -0.511 | -0.478 | -0.511 | -0.511 | -0.778 |
